# Volumetric signatures of basal ganglia–thalamo–cortical and cerebello–thalamo–cortical networks in Parkinson's disease and its motor subtypes

**DOI:** 10.3389/fnagi.2026.1743479

**Published:** 2026-02-09

**Authors:** Fatemeh Sadeghi, Abdullah Okar, Ronak Rashedi, Christian Gerloff, Dagmar Timmann, Robert Schulz, Simone Zittel

**Affiliations:** 1 Department of Neurology, University Medical Center Hamburg-Eppendorf, Hamburg, Germany; 2 Institute of Computational Neuroscience, Hamburg Center of Neuroscience, University Medical Center Hamburg-Eppendorf, Hamburg University, Hamburg, Germany; 3 Department of Neurology and Center for Translational Neuro- and Behavioral Sciences (C-TNBS), Essen University Hospital, Essen, Germany

**Keywords:** basal ganglia–thalamo–cortical network, biomarkers, cerebello–thalamo–cortical network, machine learning, motor subtypes, Parkinson's disease, quantitative susceptibility mapping, volumetric MRI

## Abstract

**Introduction:**

Parkinson's disease (PD) is a systems-level disorder, implicating basal ganglia–thalamo–cortical (BTC) and cerebello–thalamo–cortical (CTC) networks. While regional atrophy has been reported, network-wide volumetric profiles and their relevance for subtype classification and symptom association remain underexplored.

**Methods:**

We acquired T1-weighted MRI and quantitative susceptibility mapping (QSM) from 40 PD patients and 21 healthy control participants (HC). Volumes were extracted from 19 regions of interest (ROI) within the BTC and CTC networks using a multimodal pipeline. We assessed asymmetry, group differences, and symptom associations using regression models, and applied ridge regression models for PD vs. HC and motor subtype classification.

**Results:**

Network-level ROI volumes successfully classified PD vs. HC and PD motor subtypes, with the highest optimistic AUC of 0.88 for PD vs. HC (mean AUC of 0.63) and 0.95 for PD-TD vs. PD-PIGD (mean AUC reached 0.68). The thalamic nuclei and cerebellar lobules I–V, VIIIa, X were identified as key features. Atrophy in the dentate nucleus (DN), substantia nigra–subthalamic complex (SN–STN), and M1 predicted PD. Tremor severity correlated with the ventral lateral posterior thalamus (VLp), VIIb, and SN–STN volumes; bradykinesia severity with the thalamus; and postural instability and gait disturbance (PIGD) with lobule IV. No significant group-level differences for single volumes were found.

**Conclusion:**

Multiregional volumetric analysis within the BTC and CTC motor networks uncovered group differences between PD and HC that were not apparent when examining single ROIs alone. These findings highlight that PD-related alterations manifest as distributed volumetric patterns across interconnected motor circuits, supporting their role as imaging biomarkers.

## 1 Introduction

Parkinson's disease (PD), the second most prevalent progressive neurodegenerative disease worldwide (Poewe et al., 2017), was long considered a focal disorder of nigrostriatal dopamine loss, yet convergent neuropathological, electrophysiological and imaging evidence now depicts PD as a systems-level disturbance that propagates through interlocking motor circuit networks (Caligiore et al., 2016; McGregor and Nelson, 2019). Dopamine neuron loss originates in the substantia nigra (SN) and spreads anterogradely along the nigro-striatal pathway to the striatum, where the dopamine depletion initiates a cascade of structural and physiological alterations. Subsequent trans-synaptic degeneration then advances to basal-ganglia output nuclei and their cortical and sub-cortical targets, driving iron-mediated oxidative stress and, ultimately, regional volume loss—a progression documented by post-mortem analyses and longitudinal MRI studies (Brettschneider et al., 2015; Halliday et al., 2008; Morris et al., 2024; Zeighami et al., 2015). Within the basal ganglia-thalamo-cortical (BTC) network, the basal ganglia output converges on the anterior ventrolateral thalamus (VLa) and projects to primary motor cortex (M1). In contrast, the cerebello-thalamo-cortical (CTC) network originates in the dentate nucleus (DN), relays via the posterior ventrolateral thalamus (VLp) and returns to M1. When volume loss or micro-architectural disruption accumulates within or between these networks, the finely tuned balance of excitatory and inhibitory drive is disturbed, and thus clinical symptoms emerge (Caligiore et al., 2016).

Recent models link the main motor phenotypes of PD to dysfunction across these interconnected motor networks rather than isolated structures. Tremor is thought to emerge from pathological interactions between the BTC and CTC networks, where aberrant pallidal firing triggers and cerebellar circuits sustain the rhythmic movements—a mechanism captured by the “dimmer-switch” theory (Helmich, 2018). Postural instability and gait disturbance (PIGD) are attributed to disrupted communication between basal ganglia output, the subthalamic nucleus (STN)–pedunculopontine complex, and cerebellar locomotor regions, impairing the network coordination required for stepping (Kim et al., 2018; Snijders et al., 2016). Bradykinesia is linked to over-recruitment of the hyperdirect pre-SMA/SMA and STN pathway, suppressing thalamo-cortical output, with cerebellar engagement viewed as a partial compensatory mechanism (Bologna et al., 2020; Sarasso et al., 2024). Together, these models emphasize PD as a disorder of large-scale motor networks and motivate a distributed structural investigation (Basaia et al., 2022).

Volumetric MRI offers a structural correlate of such network-wide alterations. T1-weighted (T1-w) morphometry has revealed atrophy patterns across basal ganglia regions, cortical thinning in M1 and SMA, and lobule-specific cerebellar volume loss even in early PD (Jia et al., 2019; Kerestes et al., 2023; Lin et al., 2017; Poston et al., 2020). However, T1-w contrast is suboptimal for iron-rich nuclei—including substantia nigra (SN), STN, red nucleus (RN) and DN—that are critical hubs of the BTC and CTC networks. Quantitative susceptibility mapping (QSM) complements T1-w imaging by exploiting gradient-echo phase to quantify magnetic susceptibility, thereby delineating iron-loaded structures with high fidelity (Deistung et al., 2017; Eskreis-Winkler et al., 2017; Harada et al., 2022). A multimodal approach of combining T-1w and QSM images, therefore, enables simultaneous assessment of cortical, thalamic and deep-nuclear architecture.

Machine learning (ML) techniques, particularly classification models, have become indispensable in the field of neurodegenerative research, offering robust frameworks for predicting disease status, stratifying subtypes, and identifying biomarkers from complex imaging and clinical datasets. Particularly in PD, supervised classifiers such as logistic regression, support vector machines, and ensemble methods have enabled researchers to delineate patterns in multimodal data that distinguish PD patients from healthy control participants (HC) or differentiate between clinical subtypes. These models leverage volumetric features to enhance diagnostic precision, support early detection, and guide personalized therapeutic strategies (Du et al., 2024; Hwang et al., 2025; Rabie and Akhloufi, 2025).

Comprehensive, network-wide volumetric studies that integrate both BTC and CTC circuits with clinical subtyping as well as individual symptom manifestations are critical for advancing our understanding of PD (Espay et al., 2020; Miller and O'Callaghan, 2015; Schapira, 2013). Such anatomically detailed assessments can reveal early structural alterations underlying distinct symptom profiles and support the development of more specific biomarkers for diagnosis, stratification, and targeted intervention (Lotankar et al., 2017). While numerous studies have reported regional volumetric alterations in PD, most have focused on isolated regions or individual symptom domains. Even network-level investigations tend to examine either the BTC or the CTC circuit in isolation, without integrating both systems. Moreover, few studies have reported the classification power of multi-network structural changes to distinguish PD from HC and differentiate between tremor-dominant PD patients (PD-TD) and PIGD patients (PD-PIGD).

In the present study we quantified volumes of key regions within the BTC and CTC networks prospectively in early-stage PD patients, defined by the Hoehn and Yahr (HY) disease stage, and matched HCs by combining T1-w imaging for cortical and thalamic segmentation with high-resolution QSM for precise delineation of iron-rich subcortical nuclei. We investigated whether (i) PD patients exhibit distributed, network-specific volumetric alterations relative to controls; (ii) multivariate region of interest (ROI) profiles can classify PD from healthy individuals and further distinguish PD-TD from PD-PIGD patients; and (iii) network-specific structural patterns correlate with MDS-UPDRS III subscores for tremor, bradykinesia, and PIGD symptom severity. By integrating complementary MRI modalities with a network-informed segmentation framework, this study aims to deepen our understanding of the structural substrates underlying PD motor phenotypes and symptoms as well as supporting the development of network-level imaging biomarkers.

## 2 Methods

### 2.1 Participants

Twenty-two PD-TD and 21 PD-PIGD patients were recruited from the Movement Disorders Clinic at the University Medical Center Hamburg-Eppendorf between 2021 and 2024, along with 21 age- and sex-matched healthy controls (HC). All participants met the following inclusion criteria: (1) age above 18 years, and (2) written informed consent to participate in the study. Specific inclusion criteria for the patients included diagnosis of PD in accordance with the United Kingdom Parkinson's Disease Society Brain Bank (UKPDSBB) (Gibb and Lees, 1988) by a board-certified neurologist specialized in movement disorders.

Exclusion criteria applied uniformly across all participants encompassed: (1) coexistence of other neurologic disorders, including secondary or atypical PD, (2) history of deep brain stimulation or other significant head surgery, (3) electronic or metal implants, such as cardiac pacemakers and aneurysm clips, (4) pregnancy, (5) intake of other centrally acting medication such as antidepressants, (6) incompatibility with MRI safety standards, and (7) inability to provide informed consent.

### 2.2 Clinical evaluation

Standardized motor symptom evaluation of PD patients was performed based on the Movement MDS-UPDRS parts II and III in a single session (Goetz et al., 2008). The examinations were carried out by movement disorder-trained medical personnel and videos were later reviewed by a board-certified neurologist who is specialized in movement disorders as quality control. Both the examiner and the reviewing physician were blinded to the MRI data. The ratio of MDS-UPDRS subscores for tremor severity to those for PIGD was computed and used to determine clinical subtypes of PD: PD-TD and PD-PIGD (Stebbins et al., 2013). Moreover, we focused on six key symptom domains derived from MDS-UPDRS Part III: rest tremor (items 3.17–3.18), postural tremor (item 3.15), kinetic tremor (item 3.16), limb bradykinesia (items 3.4–3.8), PIGD (items 3.9–3.12), and the total motor score (MDS-UPDRS-III), which reflects the sum of all Part III items.

All patients were evaluated in the “OFF” state i.e. not having taken medication for at least 12 h prior to the experiment. To assess the influence of the daily intake of antiparkinsonian drugs the levodopa equivalent daily dose (LEDD) was calculated for each patient (Jost et al., 2023). Additionally, the handedness of participants was determined by the Edinburgh Handedness Inventory (Oldfield, 1971). The staging of PD functional disability was further identified through the HY staging (Hoehn and Yahr, 1967). This study has been approved by the local ethics committee of Hamburg (2020-10281_BO-ff). All participants provided written informed consent, and the study was performed in accordance with the ethical standards as laid down in the Declaration of Helsinki.

### 2.3 MRI processing and volumetry analysis

#### 2.3.1 MRI image acquisition

All participants underwent identical MRI scanning sequences using a 3T Prisma Siemens scanner (Siemens Healthineers, Erlangen, Germany) at the Department of Neuroradiology at the University Medical Center Hamburg-Eppendorf. For all scans, 64-channel head array coils were used. To minimize MRI artifacts from head motion, particularly in PD participants with tremor, a snug head coil and stabilizing cushions were used to further secure the head. T1-w MRI images were acquired using a magnetization-prepared rapid gradient-echo (MPRAGE) sequence, resulting in 256 coronal slices with a field of view (FOV) = 230 mm, repetition time (TR) = 2,500 ms, echo time (TE) = 2.15 ms, flip angle = 8°, voxel size = 0.8 × 0.8 × 0.8 mm, matrix dimension = 232 × 288 × 256, scanning time = 5′:49^′′^, and bandwidth = 240 Hz/pixel.

QSM images were obtained using a multi-echo, 3D gradient echo (GRE) sequence. Acquisition parameters included: FOV = 230 mm with a phase FOV of 81.3%, TR = 50 ms, TE = [3.02, 7.35, 12.23, 17.11, 21.99, 26.87, 31.75, 45.00] ms, flip angle = 20°, voxel size = 1.2 mm × 1.2 mm × 1.2 mm, matrix dimension = 232 × 288 × 256, scanning time = 5′:49^′′^, bandwidth = [370, 260] Hz/pixel. The acquisition used a 192 × 100% base and phase resolution with a partial Fourier acquisition of 7/8. The imaging was conducted using a GRAPPA (Generalized Autocalibrating Partially Parallel Acquisitions) acceleration factor of 2 for parallel imaging, with 24 reference lines in the phase encoding direction.

#### 2.3.2 T1-w image preprocessing

After an initial visual inspection of T1-w images to ascertain their quality, they were preprocessed using the recon-all pipeline in FreeSurfer (v7.1; Desikan et al., 2006; Fischl, 2012). The process performs skull stripping, intensity normalization, and anatomical segmentation based on the Desikan-Killiany atlas (Alexander et al., 2019). Since motion-related artifacts are common in PD, especially due to tremor, a trained examiner manually reviewed all segmentations and applied slice-by-slice corrections following FreeSurfer's quality control protocols.

#### 2.3.3 QSM processing

QSM images were processed using the SEPIA toolbox (v1.0; Chan and Marques, 2021). Phase and magnitude data were preprocessed per subject session, beginning with brain extraction using iBET with optimized fractional and gradient thresholds. Phase unwrapping was performed using a Laplacian-based method within the MEDI algorithm (Liu et al., 2011) with echo combination based on optimal weighting. Background field removal was conducted using RESHARP (Sun and Wilman, 2014) with an erosion radius of 0 and α = 0.01. QSM maps were reconstructed using MEDI (Liu et al., 2012), referencing cerebrospinal fluid (CSF) as the baseline tissue. Parameters included a regularization value (λ) of 1,000 and a 90% phase mask threshold. Spatial smoothing was applied using a spherical mean value (SMV) filter with a radius of 5 voxels. All QSM images were registered to their corresponding T1-w images to ensure consistent anatomical alignment between modalities.

#### 2.3.4 Segmentation and volume extraction

A total of 19 ROIs were selected based on their relevance to the BTC and CTC networks, and their volumes were extracted for subsequent analyses. These included the SN–STN complex, the thalamus (total volume, as well as VLa and VLp), M1, the DN, the whole cerebellum, and 12 cerebellar lobules (I–X, Crus I–II). Both, the thalamus and M1 were considered components of both BTC and CTC circuits. Furthermore, the intracranial volume (ICV) was extracted to account for variation in head sizes during subsequent analyses.

##### 2.3.4.1 Automated segmentation of cortical and subcortical structures

The thalamus was automatically segmented on T1-w images using the Connectome Mapper pipeline (v3.1; Tourbier et al., 2022) in conjunction with the Lausanne 2018 atlas (Najdenovska et al., 2018), which provides fine parcellation of thalamic sub-nuclei, including VLa and VLp. The M1 was defined anatomically as the precentral gyrus based on the Desikan-Killiany atlas and segmented using FreeSurfer (Desikan et al., 2006).

##### 2.3.4.2 Cerebellar segmentation

Cerebellar volumes were obtained via the CERES pipeline (Carass et al., 2018; Romero et al., 2017), which accounts for individual variability in age and sex. This provided volumetric estimates of cerebellar hemispheres and lobules. These segmentations were integrated into the full image processing pipeline alongside other ROIs.

##### 2.3.4.3 Manual segmentation on QSM images

All deep nuclei and cerebellar lobules not covered by automated pipelines were manually segmented on QSM–T1 registered images using ITK-SNAP (v4.0; Yushkevich et al., 2006). Segmentation was performed on pseudonymized MRI images in 3D by a trained rater blinded to all clinical information. To ensure segmentation reliability, each ROI was independently re-segmented by a second experienced rater who was also blinded to subject identifiers. The delineation criteria, segmentation steps, and review procedures were discussed and agreed upon by both raters prior to analysis. All masks were visually verified to exclude outliers. Inter-rater reliability was further quantified using the Intraclass Correlation Coefficient (ICC), calculated from a two-way random effects model to assess measurement consistency across raters (Koo and Li, 2016; Shrout and Fleiss, 1979). This model allowed generalization beyond the raters and samples included in the present study.

Segmentation of the SN–STN complex followed established anatomical landmarks based on QSM contrast. The medial border was defined by the RN, the lateral edge by the internal capsule, and the superior boundary was aligned with the dorsal limit of the STN. The RN itself was identified as a round midbrain structure located posterior to the SN, and was delineated according to its known spherical appearance (Manova et al., 2009; Poston et al., 2020). Due to the anatomical proximity and overlapping susceptibility profiles of the SN and STN properties (de Hollander et al., 2017; Plantinga et al., 2014), as well as variability in nigrosome 1 visibility within the SN (Cheng et al., 2020), both regions were segmented as a combined ROI. This approach prioritized robust volume quantification over exact sub-structure separation. Segmentation was guided by previous protocols developed for high-resolution QSM datasets above 3T (Deistung et al., 2013; Poston et al., 2020).

The DN, identifiable on QSM by its high iron content and laminated architecture (Deistung et al., 2022; Koeppen, 1995), appears distinctly hyperintense relative to surrounding cerebellar tissue. To enhance contrast and improve border visibility, relative contrast thresholding was applied in ITK-SNAP (Yushkevich et al., 2006), with manual adjustment of brightness parameters to optimize the intensity histogram and reveal the DN's characteristic serrated edges. Segmentation was initiated in the axial plane at the slice where boundaries were most clearly defined, then extended into sagittal and coronal views to ensure full spatial coverage.

All segmentations were reviewed and refined for anatomical precision. Final ROI volumes were calculated in mm3 and normalized by ICV to account for inter-individual differences in head size.

### 2.4 Overview of statistical analysis

All statistical analyses were conducted using R (version 4.3.2) and Python (version 3.8.12). A significance threshold of *p* < 0.05 was applied throughout. Where applicable, *p*-values were adjusted for multiple comparisons using the false discovery rate (FDR) correction (Benjamini and Hochberg, 1995). For classification algorithms and models associating volume with symptom severity, leave-one-out cross-validation (LOOCV) was used to estimate *p*-value stability, and median *p*-values across iterations were reported.

#### 2.4.1 Cohort demographics and data characteristics

We assessed demographic and clinical differences between groups (HC, PD-TD, PD-PIGD) using *t*-tests (for age and HY), Fisher's exact test (for sex) and Mann–Whitney U test (for LEDD). Normality of volumetric data was tested using Shapiro–Wilk tests (Shapiro and Wilk, 1965) considering our sample size; and homogeneity of variance was assessed using Levene's test (Levene, 1960) for its robustness to non-normal data distribution.

#### 2.4.2 Anatomical and clinical asymmetry

To quantify hemispheric volume differences, we computed the anatomical asymmetry index (*AI*_*anatomical*_) for each ROI as described by (Kurth et al., 2015).


$$\begin{array}{l} AI_{anatomical}=\;\frac{Right-Left}{Right+Left} \end{array}$$ (1)

For evaluating volumetric asymmetry in relation to clinical symptoms, we determined symptom lateralization by comparing right and left MDS-UPDRS Part III scores, designating the more affected side (MAS) and less affected side (LAS) for each patient. ROIs were then assigned contralaterally, except for cerebellar regions and the DN, which were assigned ipsilaterally. For HC and in cases of symmetric symptom manifestation, the average of left and right ROI volumes was considered for both MAS and LAS. The clinical asymmetry index (*AI*_*clinical*_) was calculated as below.


$$\begin{array}{l} AI_{clinical}=\;\frac{MAS-LAS}{MAS+LAS} \end{array}$$ (2)

One-sample *t*-tests evaluated whether AI values significantly differed from zero. Group-level differences in both asymmetry measures were assessed using ANCOVA; with age, sex, HY stage, and handedness included as covariates. To explore the relationship between structural and clinical asymmetry, Kendall correlation analyses were performed across ROIs.

#### 2.4.3 Group differences in regional volumes

Normalized ROI volumes were compared across cohorts using linear regression models. Separate models were constructed for MAS and LAS volumes (with *AI*_*clinical*_), with age and sex included as covariates (HY stage was added for PD subtype comparisons). Performance of all models were tested by repetition after outlier exclusion using the median absolute deviation (MAD) method (Leys et al., 2013).

#### 2.4.4 Cohort classification using motor network volumes

To examine whether regional brain volumes contain cohort-specific structural information (PD vs. HC; PD-TD vs. PD-PIGD), we applied multivariate classification models. Analyses were performed using z-scored MAS volumes to ensure feature comparability among large and small ROIs, and to prevent disproportionate influence from high-variance regions. Multicollinearity between ROI volumes was addressed by computing pairwise correlations and removing redundant features with *r* > 0.8.

Classification was conducted using ridge regression with 80 to 20 random train-to-test splits, iterated over 500 subsamples to obtain stable and unbiased performance estimates (Kohavi, 1995). Each train-test split was generated using random sub-sampling while preserving class balance between PD and HC groups in each iteration. Data preprocessing (including *z*-scoring) was performed independently within each training set to avoid data leakage. Ridge regression models were implemented using the *glmnet* framework with alpha = 0 (pure L2 regularization). The regularization strength (λ) was optimized within each training set using 5-fold cross-validation (*cv.glmnet*) over a fixed grid of candidate values [λ = 10^∧^seq(−4, 2, length = 100)], and the value minimizing cross-validated error (*lambda.min*) was used for model fitting and prediction. Additionally, LOOCV was used to evaluate generalizability. As a benchmark, a dimensionality-reduced model using principal component analysis (PCA) was implemented, extracting the top 10 principal components, followed by logistic regression on identical training splits, allowing comparison of performance and interpretability.

Models were trained both on all ROIs combined, and on each ROI individually. This dual strategy enabled assessment of both multivariate feature importance and univariate discriminative power via individual area under the curve (AUC). Feature importance reflects a region's contribution in the context of all features, while individual AUC quantifies its stand-alone predictive value. Model performance was summarized as the mean ± SD and 95 % confidence intervals of AUC across 500 iterations. Additionally, the mean AUC of the top 10 % best-performing iterations was reported as an optimistic performance indicator.

#### 2.4.5 Association between motor network volumes and symptom severity

Multiple linear regression models were used to assess whether ROI volumes were associated with motor symptom severity (tremor, bradykinesia, PIGD, and MDS-UPDRS-III scores). In the multi-ROI analyses, all predefined ROIs within the BTC and CTC networks were entered simultaneously as independent variables in a single model, without stepwise or data-driven feature selection, to capture distributed network-level associations. Separate models were constructed for each combination of ROI and symptom, adjusting for age, sex, and HY. Analyses were performed using *z*-scored MAS volumes to standardize input scales across regions. Robustness of model significance was evaluated using LOOCV and the median *p*-value across all iterations was reported. In parallel, single-ROI models were fitted for each region individually to quantify stand-alone associations and facilitate interpretability.

## 3 Results

### 3.1 Cohort demographics and data characteristics

Two patients were excluded due to the insufficient quality of the acquired MRI images for processing. Additionally, one patient and two HC were excluded due to coincidental abnormal MRI findings. Table 1 summarizes the demographic information of the final study cohort. The HC group had a mean age of 63 ± 9 years (58% females); the PD-PIGD cohort 68 ± 8 years (female ratio: 39%), and the PD-TD cohort 61 ± 9 years (female ratio: 41%). No significant age difference was observed between HC and PD groups (*t*-test, *p* = 0.838), nor was there a difference in sex distribution (Fisher's exact test, *p* = 0.266). However, a significant age difference was found between PD motor subtypes, with PD-TD patients being younger than PD-PIGD patients (*p* = 0.010); we therefore consistently included age as a covariate in all analyses and interpreted age-sensitive findings with caution. In addition, we performed an age-matched sensitivity analysis for PD-TD vs. PD-PIGD single-ROI comparisons, which showed preserved effect directions and no contradictory findings relative to the main analysis (see Supplementary Table S1). No significant differences were observed between PD-TD and PD-PIGD groups in sex distribution (*p* = 1.000) or HY stage (*p* = 0.496). LEDD values did not differ significantly between PD-TD and PD-PIGD groups (Mann–Whitney *U* = −92, *p* = 0.321), suggesting that medication dosage is not a major confounding factor for the observed group differences.

**Table 1:** Demographics and clinical characteristics.

**Demographics**	**HC (*n* = 19)**	**PD total (*n* = 40)**	**PD-TD (*n* = 22)**	**PD-PIGD (*n* = 18)**
Age (y)	63 [50–89]	64 [44–84]	61 [44–76]	68 [54–84]
Sex (female/male)	11/8	16/24		
Disease duration (y)		4.47 [0–13]	3.36 [0–12]	5.83 [2-13]
LEDD		572.28 [0–1364]	522.99 [0–1288]	632.52 [190–1364]
Hoehn and Yahr		Stage 2: *n* = 35	Stage 2: *n* = 20	Stage 2: *n* = 15
		Stage 3: *n* = 5	Stage 3: *n* = 2	Stage 3: *n* = 3
Handedness (right/left/ambidextrous )	18/1/0	37/1/2	19/1/2	18/0/0
Most affected side (right/left/equal)		20/17/3	14/7/1	6/10/2
MDS-UPDRS III		30.02 [12–54]	31.05 [13–54]	28.78 [12–45]
Tremor		6.38 [0–18]	9.14 [2–18]	3.00 [0–10]
Resting tremor		3.15 [0–12]	5.09 [0–12]	0.78 [0–4]
Postural tremor		1.30 [0–4]	1.55 [0–4]	1.00 [0–4]
Kinetic tremor		0.70 [0–3]	0.82 [0–3]	0.56 [0–2]
Bradykinesia		14.65 [5–29]	14.64 [5–29]	14.67 [5–26]
PIGD		1.95 [0–11]	1.23 [0–5]	2.83 [0–11]

Values are presented as mean [minimum–maximum] unless otherwise indicated. MDS-UPDRS, movement disorders society unified Parkinson's disease rating scale; HC, healthy controls; PD, Parkinson's disease patients; TD, tremor-dominant; PIGD, postural instability and gait disturbance; LEDD, levodopa equivalent daily dose.

### 3.2 Volumetry analysis

**Figure 1:**
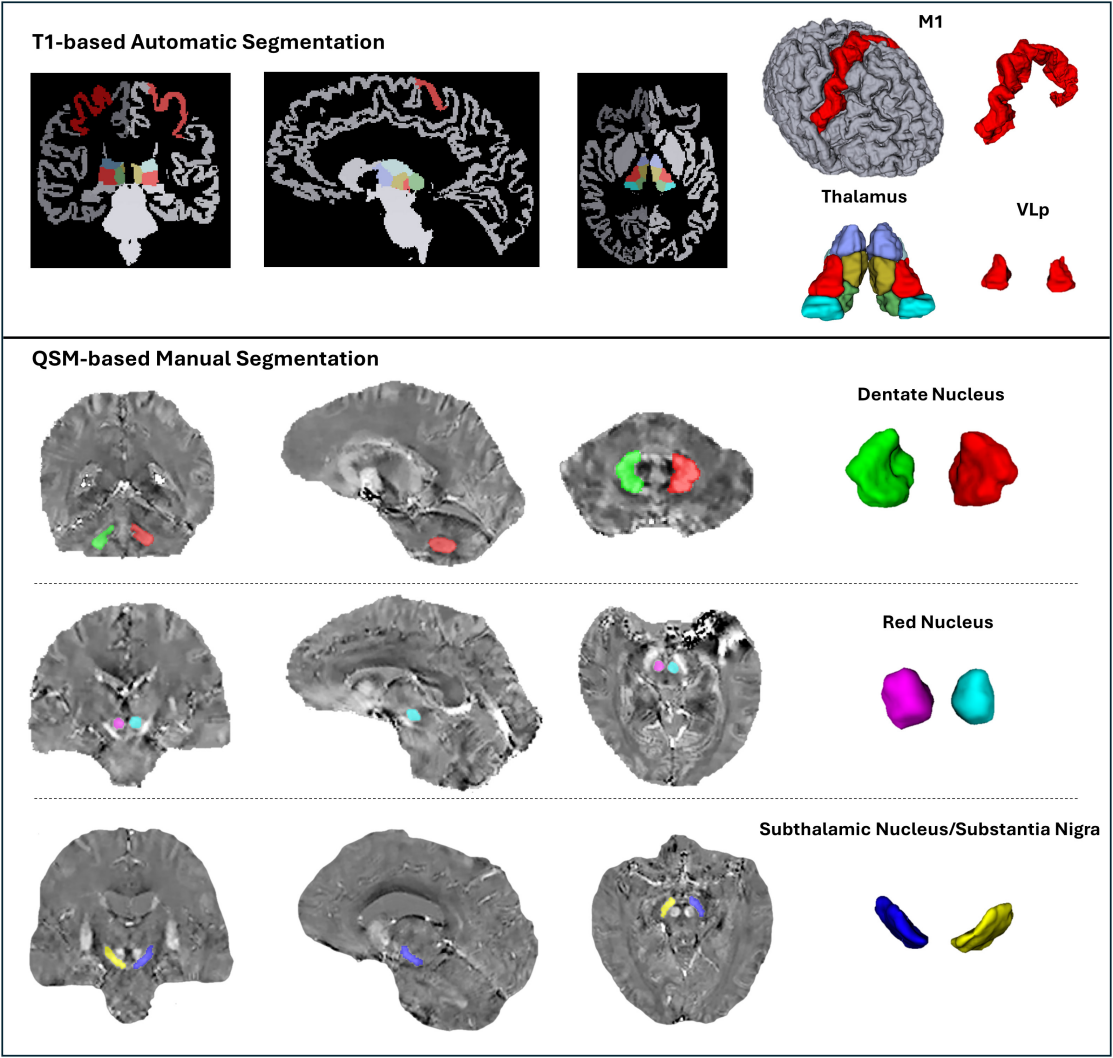
Representative example of ROI segmentations used for volumetry analysis. **Top row**: Automated segmentation of thalamus, M1 (precentral gyrus), and cerebellar lobules using FreeSurfer, the Connectome Mapper pipeline, and CERES. **Middle and bottom rows**: manual segmentation of deep nuclei on QSM–T1 co-registered images, including the DN and SN–STN complex. The 3D reconstructions of selected ROIs are shown for visual clarity. All segmentations were performed in native space and verified across multiple planes.

All automated and manual segmentations were successfully completed across participants without major artifacts or processing failures. The extracted regional volumes served as the basis for subsequent statistical analyses. A representative example of the anatomical segmentation results is shown in Figure 1, including both cortical and subcortical regions from the BTC and CTC networks. Levene's tests revealed no significant violations of variance homogeneity across cohorts for any of the volumetric measures (all *p* > 0.05). Similarly, Shapiro–Wilk tests indicated that most ROI volumes conformed to normality (*p* > 0.05), with only mild deviations in certain smaller regions such as lobule IX, which upon closer inspection were deemed acceptable for parametric analysis. Detailed plots are provided in the Supplementary Figure S1.

### 3.3 Anatomical and clinical asymmetry

We observed significant anatomical asymmetry in a number of regions. Rightward asymmetry was observed in the cerebellum (HC: *AI*_*anatomical*_ = 0.006; PD-TD: 0.009; PD-PIGD: 0.005; all FDR *p* < 0.001), Crus I (HC: *AI*_*anatomical*_= 0.011; PD-TD: 0.017; PD-PIGD: 0.022; all FDR *p* < 0.01), Crus II (HC: *AI*_*anatomical*_= 0.019; PD-TD: 0.031; PD-PIGD: 0.019; all FDR *p* < 0.01), and VLp (PD-TD: *AI*_*anatomical*_= 0.036, FDR *p* < 0.001), with similar patterns across groups. In contrast, leftward asymmetry was found in the DN (PD-PIGD: −0.084, FDR *p* < 0.001), RN (PD-TD: −0.044, FDR *p* < 0.01), thalamus (PD-TD: −0.026, FDR *p* < 0.001), and VLa (PD-TD: −0.018, FDR *p* < 0.001). The remaining ROIs showed no significant asymmetry (all FDR *p* > 0.05).

The ANCOVA analyses (controlled for age, sex, HY stage, and handedness) showed that these asymmetry patterns were not significantly different between cohorts (all FDR *p* > 0.05), suggesting cohort-independent anatomical asymmetry. Furthermore, no significant effects of handedness were observed across regions (all FDR *p* > 0.05).

One-sample *t*-tests revealed that none of the clinical asymmetry indices (*AI*_*clinical*_) significantly deviated from zero (all FDR *p* > 0.05), indicating the absence of systematic lateralization of volumes across the motor networks due to symptom expression. Furthermore, no significant alignment was observed between anatomical and clinical asymmetry, as confirmed by Kendall correlation analyses across all ROIs (all FDR *p* > 0.05). Group comparisons between PD subtypes revealed no significant differences in clinical asymmetry across any region (all FDR *p* > 0.05). Importantly, no significant associations were found between anatomical and clinical asymmetry (all FDR *p* > 0.05), or between asymmetry indices and handedness (all FDR *p* > 0.05). Detailed plots are provided in the Supplementary Figures S2, S3.

Therefore, for all subsequent analyses involving patient data, MAS ROIs were included based on clinical lateralization; while for HC, the average of both hemispheres was used. Anatomical asymmetry and handedness were not considered further, as neither was found to systematically influence the results.

### 3.4 Group differences in regional volumes

**Figure 2:**
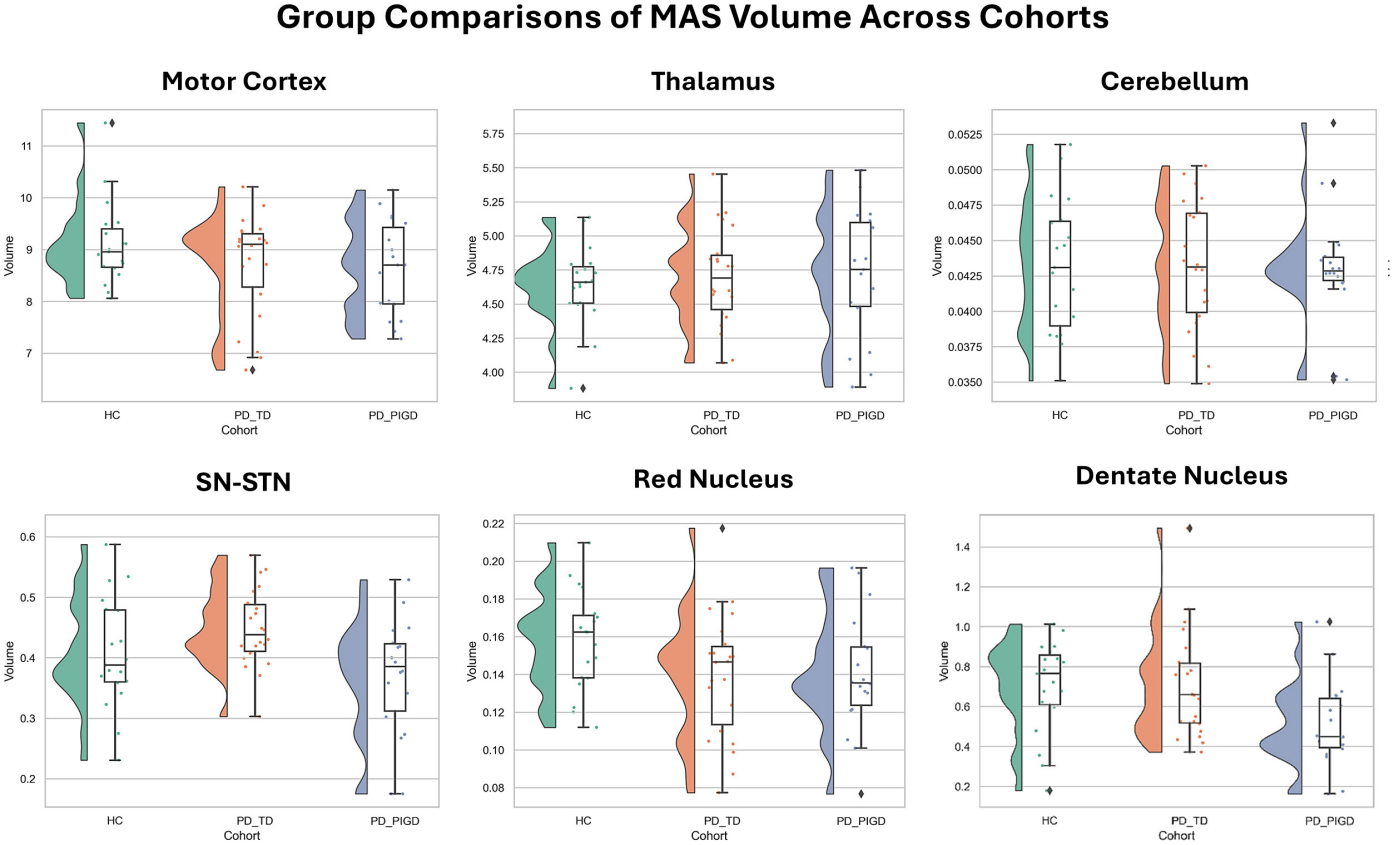
Group Comparisons of more affected side (MAS) volume across cohorts. Violin plots with embedded boxplots showing more affected side (MAS) volume across healthy controls (HC), tremor-dominant (PD-TD), and postural instability/gait difficulty (PD-PIGD) subtypes for a selection of six representative regions: motor cortex, thalamus, cerebellum, substantia nigra–subthalamic area (SN–STN), red nucleus, and dentate nucleus. No significant group differences were observed in any region (all FDR-corrected *p* > 0.05). Boxplots display the median and interquartile range, with whiskers and individual outliers overlaid.

No significant group differences in MAS volume were observed between PD patients and HC in any region (all FDR *p* > 0.05). These results remained consistent after outlier removal. As shown in Figure 2, volume distributions overlapped substantially across groups, reinforcing the absence of robust group-level effects.

### 3.5 Cohort classification using motor network volumes

No multicollinearity was detected among the ROI volumes, as verified by pairwise correlation analysis (see Supplementary Figure S4). Ridge regression and PCA yielded highly comparable classification performance across all tasks, with similar mean AUCs (ridge: 0.63 ± 0.13, 95 % CI = 0.62–0.64; PCA: 0.64 ± 0.13, 95 % CI = 0.63–0.65). Given this parity in predictive accuracy, we prioritized ridge regression for further interpretation due to its enhanced model transparency and more consistent feature importance estimates across splits and iterations.

**Figure 3:**
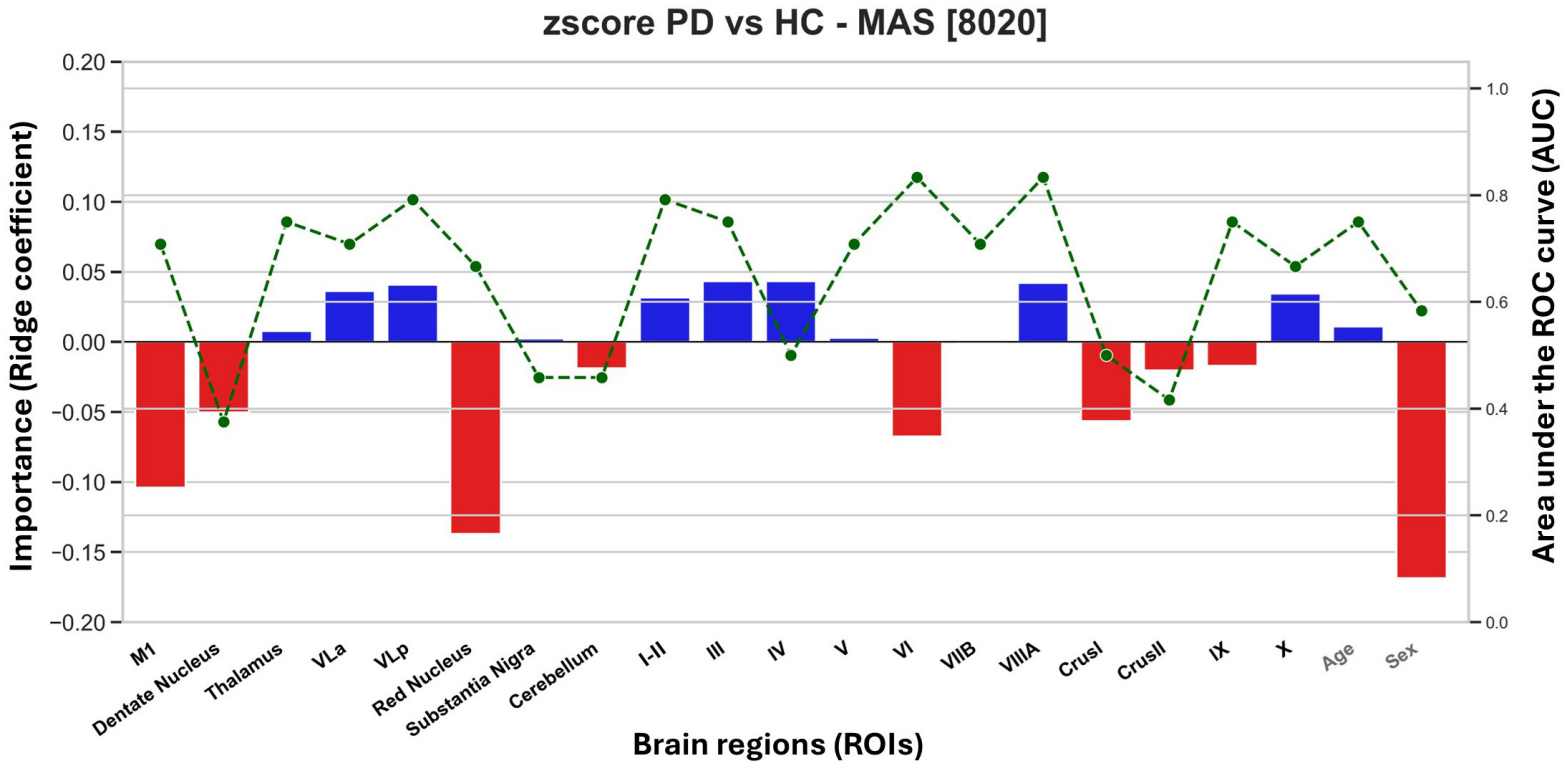
Feature importance and individual-region area under the curve (AUC) for Parkinson's disease (PD) vs. healthy controls (HC) classification using motor network volumes. Bar heights reflect ridge regression importance values (positive: preserved volume predictive of PD; negative: degeneration predictive of PD). Overlaid green lines indicate AUCs from single-region classifiers, complementing the multivariate model by showing discriminative power of individual regions of interest (ROI).

Using *z*-scored MAS volumes, classification of PD vs. HC reached an average AUC of 0.88 and accuracy of 0.86 among the best-performing iterations, while the overall mean across 500 iterations was 0.63 ± 0.13 (95 % CI = 0.62–0.64). Feature-wise AUCs and importance scores are shown in Figure 3.

Feature importance analysis revealed that the strongest positive contributors to classification included the thalamus, VLa, VLp, and cerebellar lobules I–V, VIIIa, and X. These regions showed positive weights in the model, suggesting that preserved or increased volume in these areas supports PD classification. Conversely, regions such as M1, DN, RN, cerebellum (as a whole), and lobules VI, Crus I-II, and IX contributed negatively, indicating that their volume reduction is informative of PD. To complement importance values, we also computed individual-region AUCs. While some regions had small model weights, they still demonstrated strong standalone discriminative power—most notably lobule VIIIa (AUC = 0.83), VLp (AUC = 0.79), I–II (AUC = 0.79), and thalamus (AUC = 0.75). Several negatively weighted regions like M1 (AUC = 0.71) and VLa (AUC = 0.83) also achieved high AUCs individually, reinforcing their relevance in PD-related volume reduction.

Covariates showed moderate predictive value. Age was weakly informative (AUC = 0.75, importance near zero). Sex showed a small but consistent directional contribution (AUC = 0.58), with male sex slightly associated with PD classification.

**Figure 4:**
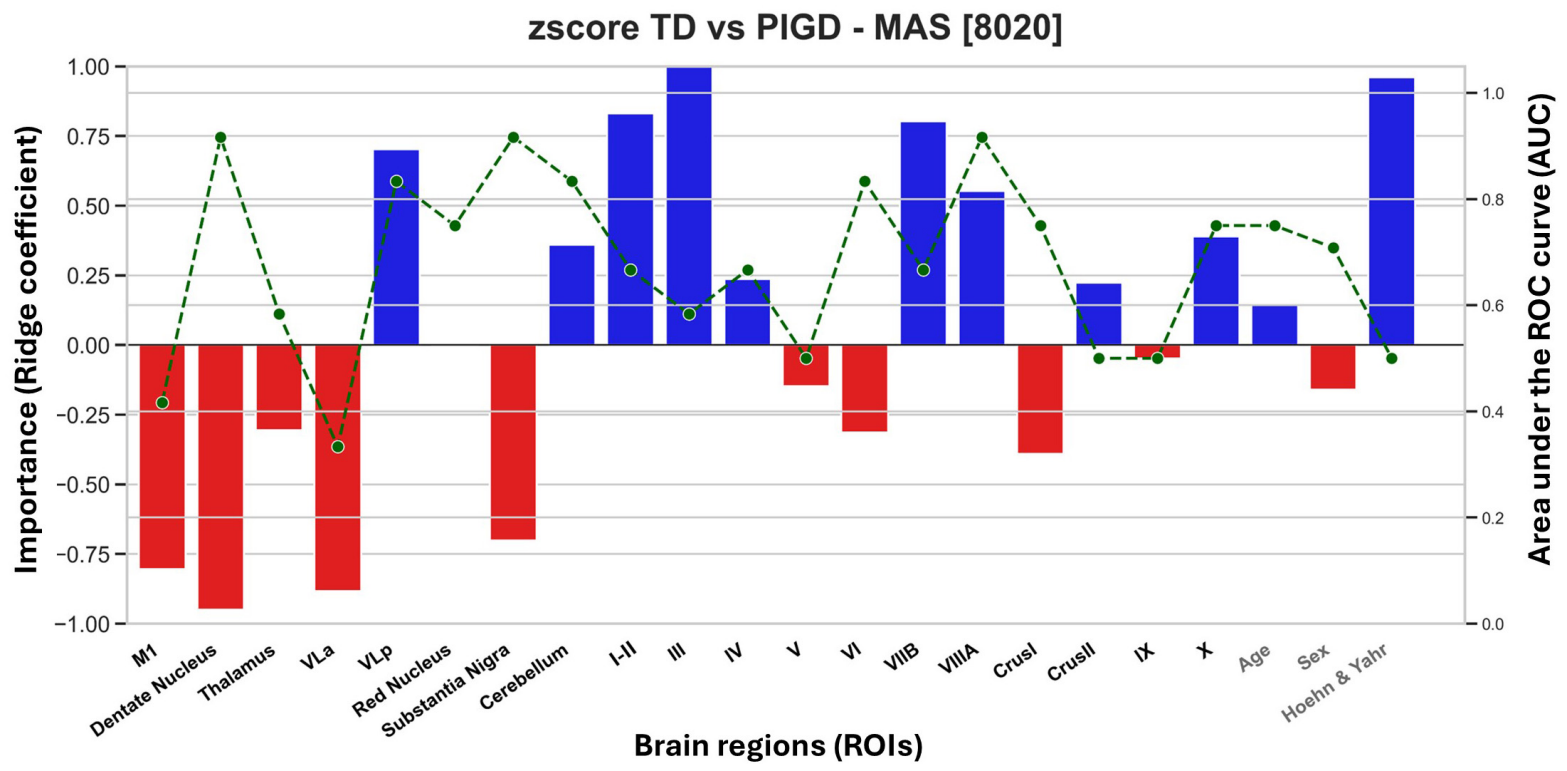
Feature importance and individual-region AUCs for PD-TD vs. PD-PIGD classification using motor network volumes. Bar heights reflect ridge regression importance values (positive: preserved volume predictive of PD-PIGD; negative: degeneration predictive of PD-TD). Green lines show AUCs from classifiers trained on single regions.

To perform motor subtype classification, the ridge model was trained to identify PD-TD participants using *z*-scored MAS volumes, with positive contributions indicating regional preservation predictive of PD-TD and negative contributions indicating reduced volumes associated with PD-TD (Figure 4). The model achieved a mean AUC of 0.66 ± 0.15 (95 % CI = 0.65–0.68) across 500 iterations, with top-performing runs reaching an AUC of 0.95 and an accuracy of 0.72. PCA produced comparable results (mean AUC = 0.67 ± 0.14, 95 % CI = 0.65–0.68; top AUC = 0.92), confirming the robustness of the multivariate classification approach. The strongest positive contributors for PD-TD included lobules I–II, III, VIIb, VIIIa, and VLp. In contrast, M1, DN, thalamus, VLa, and SN–STN showed the most negative importance scores. Individual ROI classifiers (green dotted line in Figure 4) revealed the highest AUCs (≥0.83) for VLp, SN–STN, cerebellum, and lobules VI and VIIIa. HY stage also contributed positively (importance = 0.10), while age and sex showed negligible effects. LOOCV results remained consistent (AUC = 0.67, balanced accuracy = 50%).

**Figure 5:**
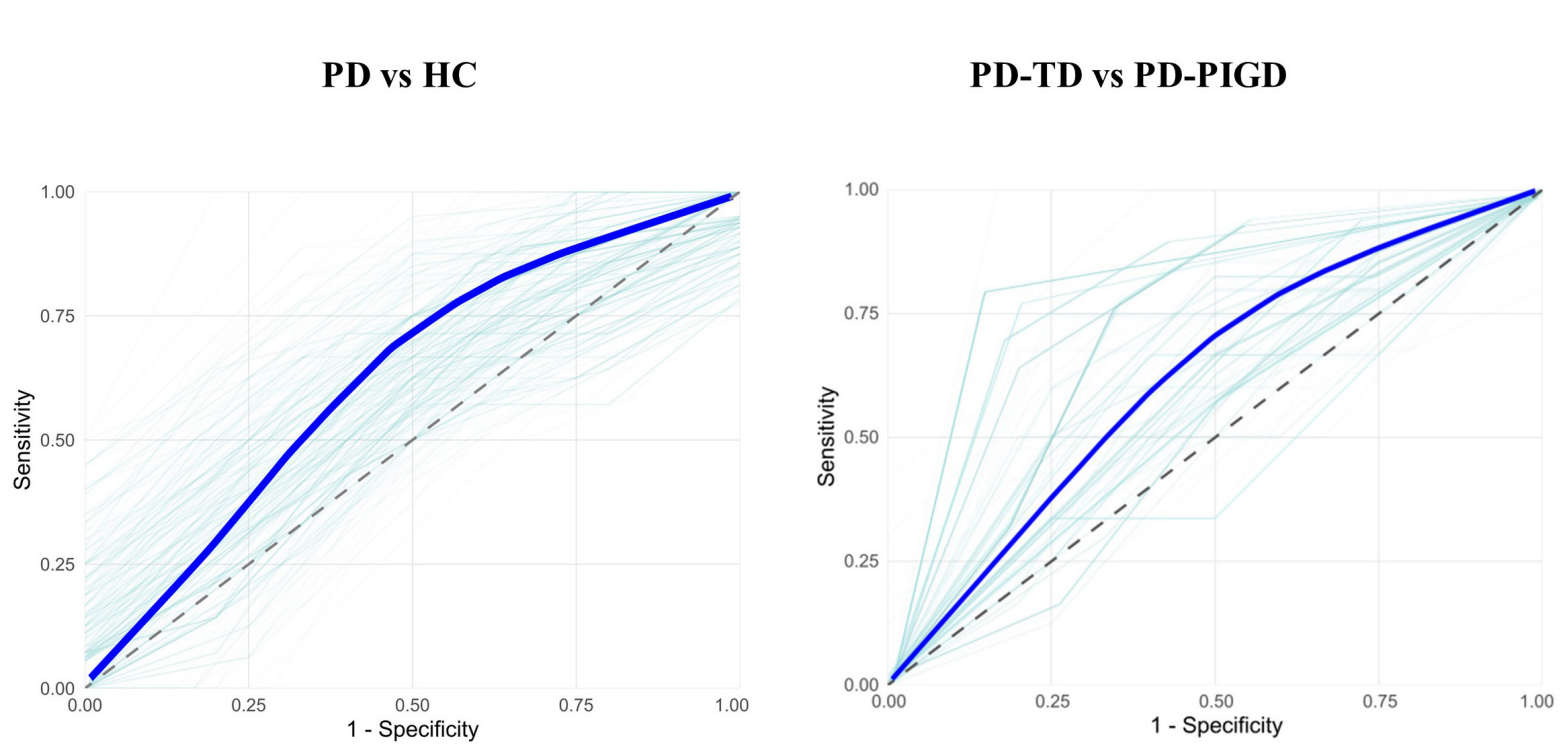
Receiver operating characteristic (ROC) curves of classification. Using ridge regression and *z*-scored more affected side (MAS) volumes across 500 iterations, the **left panel** shows the classification between Parkinson's disease (PD) and healthy controls (HC), and the **right panel** depicts the classification between tremor-dominant (PD-TD) and postural instability/gait difficulty (PD-PIGD) motor subtypes. Light green lines represent iteration-wise ROC curves, the thick blue line indicates the averaged ROC, and the dashed diagonal line denotes chance level.

To illustrate classification performance at the model level, receiver operating characteristic (ROC) curves summarizing sensitivity–specificity trade-offs across iterations are shown in Figure 5 for both PD vs. HC and PD subtype classification.

### 3.6 Association between motor network volumes and symptom severity

We examined how *z*-scored MAS volumes of the chosen regions relate to motor symptom severity using both multi-ROI and single-ROI linear regression models, adjusting for age, sex, and HY stage. In the multi-ROI models, total tremor was significantly associated with the volumes of the SN–STN, VLp, and lobule VIIb. Rest tremor was also associated with VLp, and postural tremor with M1, lobules I-II and IX. MDS-UPDRS-III total scores were significantly associated with volumes of the thalamus and the DN, while thalamus volume also showed a distinct association with bradykinesia. PIGD severity, in turn, was linked to reduced volume in Lobule IV.

The single-ROI models supported many of these findings and highlighted additional associations. For total tremor, significant contributions were again found for VLp and thalamus. Rest tremor remained associated with VLp, while kinetic tremor showed links to both VLp and lobule III of the cerebellum. PIGD severity was associated with VLa, and MDS-UPDRS-III score was associated with thalamus volume. Details of the significant results, including standardized beta coefficients, 95% confidence intervals, and LOOCV *p*-values, are summarized in Tables 2, 3 for multi-ROI and single-ROI models respectively.

**Table 2:** Significant results of multi-ROI models associating volumes of motor network regions with symptom severities.

**Symptom**	**Region**	Standardized β^*^	**95% CI**	**LOOCV *p*-value**
MDS-UPDRS III	Thalamus	−7.360	[−12.9, −1.83]	0.015
	DN	4.200	[0.85, 7.55]	0.020
Tremor	SN–STN	1.730	[0.14, 3.33]	0.042
	VLp	−3.200	[−4.23, −0.85]	0.007
	VIIB	−2.540	[−5.66, −0.75]	0.017
Rest tremor	VLp	−1.970	[−3.33, −0.6]	0.010
Postural tremor	M1	0.510	[0.03, 0.98]	0.043
	I–II	0.500	[0.10, 0.89]	0.021
Bradykinesia	Thalamus	−3.950	[−7.70, −0.19]	0.047
PIGD	IV	−1.260	[−2.17, −0.34]	0.012

CI, confidence interval; LOOCV, leave-one-out cross-validation. I-II, cerebellar lobules I and II.

^*^Covariates included age, sex, and HY stage.

**Table 3:** Significant results of complementary single-ROI models linking symptom severity and ROI volumes.

**Symptom**	**Region**	Standardized β^*^	**95% CI**	**LOOCV *p*-value**
UPDRS_III	Thalamus	−3.450	[−6.20, −0.69]	0.017
Tremor	Thalamus	−1.660	[−3.29, −0.04]	0.049
	VLp	−1.740	[−3.02, −0.45]	0.011
Rest tremor	VLp	−1.060	[−2.01, −0.10]	0.034
Kinetic tremor	VLp	−0.190	[−0.37, −0.01]	0.037
	III	−0.190	[−0.36, −0.01]	0.038
PIGD	VLa	−0.920	[−1.71, −0.13]	0.026

CI, confidence interval; LOOCV, leave-one-out cross-validation.

^*^Covariates included age, sex, and HY stage.

## 4 Discussion

### 4.1 Multivariate framework analysis reveals distributed motor-network signatures in PD

This study establishes a new perspective on early-stage PD by showing that, in the present cohort, its structural substrate is distributed, not regional. Using an integrative multivariate framework that combines volumetric measures across BTC and CTC networks, we identified coherent network-wide structural alterations that differentiate PD from HC and further separate PD-TD from PD-PIGD motor subtypes. Unlike most prior volumetric studies that focused on isolated anatomical structures (Banwinkler et al., 2022; Charroud and Turella, 2021; Mak et al., 2015; Sterling et al., 2017; Torres-Parga et al., 2025), this approach uncovers distributed, multiregional volumetric patterns, emphasizing that in this sample, early PD-related alterations manifest at the network level rather than as pronounced focal atrophy (Caligiore et al., 2016).

On the regional level, these distributed signatures point to a continuum between relative volume preservation and early volume reduction, suggesting an imbalance between compensatory and degenerative processes within BTC–CTC pathways in PD. By shifting the focus from isolated regional atrophy to network-wide volumetry, our findings propose sensitive MRI biomarkers that reflect early disease structural network alterations and provide a basis for distinguishing PD subtypes.

### 4.2 Lack of evident regional atrophy in early-stage PD

The absence of significant regional atrophy in standard cohort comparisons contrasts with the distributed volumetric differences detected by our multivariate analysis. This apparent discrepancy may reflect an early, masked phase of PD, during which large-scale network alterations emerge before overt tissue loss becomes detectable with conventional morphometry. At this stage, compensatory remodeling within interconnected motor circuits may temporarily preserve function, while age-related atrophy patterns partially obscure subtle disease-specific effects (Johansson et al., 2025; Zhu et al., 2025). It has to be considered though that given the modest sample size and early disease stage, the null univariate findings in our cohort may reflect limited power to detect subtle regional effects rather than their true absence.

Consistent hemispheric asymmetries across cohorts, but without group differences or alignment with lateralization, suggest that motor volume asymmetry reflects general anatomical variability rather than a disease-specific marker. This pattern, together with our cohort being predominantly in the HY stage 2 (PD-TD: 20/22, PD-PIGD: 15/18), supports the notion that adaptive and degenerative processes coexist in early-stage PD. Several studies reflect the same interpretation. In the large-scale Parkinson's Progression Markers Initiative (PPMI) cohort, Zeighami et al. (2015) reported only subtle gray-matter alterations in *de novo* PD (*n* = 232 PD, 117 HC), largely overlapping with healthy-aging trajectories. Longitudinal data further demonstrate that macroscopic atrophy in basal ganglia and cerebellum progresses slowly compared with earlier functional and network-level disruptions (Zarkali et al., 2024). Similarly, aging cohorts show comparably small annual subcortical volume declines (*n* = 653, >10 years MRI; Fujita et al., 2023), suggesting that early-stage PD changes remain concealed within normative variance. Studies in newly diagnosed patients (Mak et al., 2015; Sterling et al., 2017) also report null volumetric alterations despite altered connectivity, with longitudinal evidence indicating that structural changes typically become detectable only after more than 5 years of disease progression.

Together, these observations highlight that early-stage PD represents a masked phase of anatomical reorganization, in which compensatory preservation and emerging degeneration coexist within distributed networks. Detecting this phase requires analytical approaches sensitive to interregional covariance, such as the multivariate modeling employed here, which can reveal coherent, subthreshold alterations invisible to conventional volumetric statistics.

### 4.3 Network-based classification and compensatory–degenerative gradients

Our multivariate classification results demonstrate that volumetric configurations of BTC and CTC regions can robustly differentiate PD from HC and further separate PD motor subtypes, even in the absence of regional atrophy. Using ridge regression, the model achieved high AUC values in the best-performing iterations, which confirmed that distributed volumetric patterns carry diagnostic information. However, the overall mean AUCs across all iterations were more modest, suggesting that the highest model's performance may be optimistic in some cases. Comparable multivariate approaches in large cohorts reported similar or slightly lower AUCs than in our analysis (Solana-Lavalle and Rosas-Romero, 2021; Ya et al., 2022). Although PCA yielded similar accuracy, ridge regression provided greater interpretability and stability in small-sample data (Schulz et al., 2020).

Feature-weight analyses revealed two opposing gradients across motor circuits. Thalamic nuclei (VLa, VLp) and cerebellar lobules I–V, VIIIa, and X were the strongest positive contributors to PD classification, consistent with early compensatory reorganization within the CTC network (Xiong et al., 2023). Preserved volume in these regions may reflect adaptive mechanisms that maintain network efficiency despite dopaminergic loss. Supporting this interpretation, functional connectivity studies have shown increased VLp–VLa coupling with motor and prefrontal cortices in PD, suggesting early network-level adaptations that precede overt atrophy (Chen Z. et al., 2023). Similarly, lobules VIIIa and X have been implicated in motor symptom modulation via altered connectivity with cortical motor areas (Xu et al., 2019). Moreover, multivariate structural connectivity analyses including cerebellar and thalamic nodes have demonstrated high discriminative power in PD (Owens-Walton et al., 2019), reinforcing the relevance of the same regions identified here. Individual ROI analyses further confirmed their importance, with lobule VIIIa, VLp, and thalamus achieving high AUCs, underscoring their standalone diagnostic potential within the distributed motor network.

In contrast, negatively weighted regions, including M1, DN, RN, Crus I–II, and lobule IX, showed reduced volumes that contributed to PD classification as markers of early neurodegeneration. These findings converge with prior evidence that subcortical and cerebellar atrophy plays a critical role in PD-related motor dysfunction (Camacho et al., 2024; Ya et al., 2022). DN atrophy, observed even in early disease stages (based on HY score), has been linked to disrupted cerebellar connectivity and impaired motor-cognitive integration (Liu et al., 2013; Pietracupa et al., 2024). Similarly, RN alterations, though variable across studies, have been associated with motor severity and progression (Camlidag et al., 2014), while reductions in Crus II and lobule IX volumes have been reported in both motor and non-motor phenotypes (Ma et al., 2018; Ya et al., 2022). Functional imaging further supports these structural findings, showing that diminished DN–Crus I–II coupling disrupts integration between motor and cognitive cerebellar loops (Liu et al., 2013). Cortical involvement followed a similar pattern, with M1 thinning and gray-matter loss reflecting degeneration along the corticospinal tract (Wang et al., 2024). Finally, posterior cerebellar atrophy has been shown to improve PD classification accuracy in multivariate frameworks (Zhao et al., 2025), emphasizing its diagnostic value. Collectively, these negatively weighted features delineate a coherent subcortical–cerebellar degeneration pattern that complements the compensatory CTC preservation identified in our positive-weight analysis.

In the classification of PD subtypes, our ridge regression model indicated a robust separation between PD-TD and PD-PIGD based on distributed volumetric features. Feature-weight analyses revealed that preserved volume in cerebellar lobules I–II, III, VIIb–VIIIa, and VLp, as well as reduced volume in M1, DN, thalamus, VLa, and SN–STN, both contributed to PD-TD classification, indicating that distinct patterns of preservation and loss within CTC and BTC circuits jointly characterize this subtype. These patterns align with prior neuroimaging and electrophysiological studies showing subtype-specific circuit organization. Chen Y. et al. (2023) and Chen Z. et al. (2023) reported preserved anterior cerebellar lobules and enhanced cerebello-thalamic connectivity in PD-TD, while PD-PIGD showed broader thalamocortical disruption. Similarly, Park et al. (2025) found selective atrophy in lobule VIIIa but preserved thalamic volume in PD-TD, underscoring the discriminative role of CTC network regions for subtype separation.

Subcortical degeneration also contributed to subtype classification. Functional connectivity studies have shown weakened DN–VLp coupling in PD-TD (Chen Z. et al., 2023), and volumetric reductions in M1 have been linked to disrupted cortical motor integration (Wang et al., 2023). Additionally, volumetric differentiation of thalamic nuclei across subtypes has been reported in *de novo* PD patients, with PD-TD showing reductions in VLa and related intralaminar nuclei (Park et al., 2020). This supports our findings where VLa volume contributed negatively to PD-TD classification. Moreover, histopathological and imaging evidence suggests that SN degeneration follows distinct topographies across subtypes: Boonstra et al. (2022) reported preferential medial SN neuron loss projecting to the VLa and STN, while Poston et al. (2020) linked SN volume reductions to bradykinesia and rigidity but not tremor. These findings underscore the anatomical specificity of dopaminergic degeneration in PD-TD and may explain the negative weighting of SN–STN and VLa in our model. Together, our classification findings reflect a biologically plausible dissociation: cerebellar preservation in PD-TD may reflect compensatory plasticity, while atrophy in the SN–STN–VLa–M1 axis captures a core pathophysiological substrate of PD-TD.

This distributed dissociation across CTC and BTC networks reinforces that motor subtypes emerge from distinct patterns of structural imbalance rather than uniform atrophy. Network-level analyses further support this view: graph-theoretical studies demonstrate early disruptions in global topology (Fang et al., 2017) and improved classification performance when using multiregional, network-level features instead of isolated regional measures (Gratton et al., 2019). Consistent with this systems-level organization, HY stage contributed moderate predictive value (high importance, low AUC), in line reflecting its role as a coarse clinical correlate that complements network-derived structural metrics (Kaplan et al., 2022).

### 4.4 Structure–symptom relationships across motor networks

The regression models, controlling for age, sex, and HY stage, revealed that distinct motor-network nodes are associated with specific symptom severities. Tremor severity was positively associated with SN–STN volume and negatively with VLp and lobule VIIb volumes, mirroring the evidence that both BTC and CTC networks are involved in tremor generation (Dirkx and Bologna, 2022; Helmich, 2018), and specifically that lobule VIIb atrophy correlates with tremor severity (Piccinin et al., 2017; Sadeghi et al., 2023). Furthermore, functional imaging has shown that decreased connectivity between the DN and VLp is linked to increased tremor severity in PD-TD, reinforcing the role of VLp in tremor mechanism (Chen Z. et al., 2023; Sadeghi et al., 2024). Rest tremor was specifically linked to VLp volume, while postural tremor engaged the M1 and lobules I–II and IX, consistent with studies showing M1 and anterior cerebellar activation during postural stabilization (Wang et al., 2023; Zeng et al., 2019). Moreover, PD-TD patients show increased M1–cerebellum functional connectivity through VLp, potentially reflecting compensatory engagement of motor networks (Bu et al., 2024; Chen Z. et al., 2023; Wang et al., 2023). This pattern aligns with evidence that CTC interactions—particularly through VLp—are more functionally intact or hyperactive in PD-TD relative to PD-PIGD (Sadeghi et al., 2024).

Bradykinesia and total MDS-UPDRS-III scores were associated with thalamus and DN volumes, supporting mechanistic models where altered basal ganglia–thalamic output to M1 underlies bradykinesia (Berardelli et al., 2001). Ultra-high field MRI confirms that SN degeneration is selectively associated with bradykinesia-rigidity but not tremor (Poston et al., 2020). Furthermore, a recent longitudinal MRI study demonstrated that multivariate gray matter volume metrics predicted progression in bradykinesia and gait symptoms over 5–10 years, underscoring their prognostic value (Vijayakumari et al., 2023).

PIGD severity correlated with lobule IV atrophy in both multi- and single-ROI models. Lobule IV, part of the spinocerebellum, maintains strong functional connections to vestibulocerebellar regions involved in balance and postural stabilization (Snijders et al., 2016; Wang et al., 2023). Consistent with our findings, PD-PIGD patients exhibit reduced white matter integrity in cerebellar tracts such as the middle cerebellar peduncle and diminished thalamic connectivity relative to PD-TD (Bu et al., 2024).

Together, these symptom-specific volumetric profiles highlight that Parkinsonian motor features emerge from network-wide structural alterations rather than localized atrophy (Caligiore et al., 2016), and suggest that targeted assessment of these network nodes could inform personalized prognosis and circuit-based interventions (Vijiaratnam et al., 2021). Our findings may have implications for clinical decision support. Although the average classification performance was overall modest, the use of multivariate volumetric patterns demonstrates proof of concept for clinical applications. The distinct volumetric signatures between PD-TD and PD-PIGD could inform stratification in clinical trials, addressing the heterogeneity that often confounds therapeutic studies (Greenland et al., 2019).

### 4.5 Limitations

Several limitations should be considered when interpreting our findings. First, our cross-sectional design limits causal inferences about the relationship between volumetric changes and symptom progression. Longitudinal studies are needed to establish whether the identified volumetric signatures represent stable trait markers or evolve with disease progression. Second, the modest sample size, particularly for PD motor subtype comparisons in single ROI analysis, may have limited statistical power to detect subtle regional volumetric effects, and thus null univariate findings may not be interpreted as evidence for the absence of focal structural changes. This may explain the absence of significant regional atrophy in traditional group comparisons despite robust multivariate classification performance. Third, replication of these findings in larger and independent cohorts will be essential to confirm the generalizability and robustness of the observed network-level volumetric patterns. Fourth, our focus on motor network regions, while hypothesis-driven, may have overlooked contributions from other brain areas. Non-motor symptoms, which are prevalent even in early PD, were not consistently assessed, limiting our understanding of the full spectrum of brain-behavior relationships in our cohort. Finally, cognitive status and mood data were not available for analysis in this cohort, which limits the ability to examine their potential influence on volumetric measures and motor phenotype. These factors should be considered in future studies.

Despite these limitations, our multivariate approach successfully identified network-level volumetric signatures that robustly classified PD patients and revealed association with severity of motor symptoms, suggesting that these patterns capture biologically meaningful disease-related changes.

### 4.6 Conclusion

To conclude, this study demonstrates that multivariate modeling across PD motor network reveals meaningful, disease-relevant alterations even when regional volumetric differences may not be detectable in early-stage patient using traditional univariate analyses. By applying regularized classification techniques to ROI volumes within the BTC-CTC networks, we achieved robust discrimination of PD from HC and identified distinct volumetric profiles predictive of PD motor subtypes. These findings highlight the value of network-level structural biomarkers—particularly within the CTC circuit—as early and subtype-sensitive indicators of PD. Importantly, the convergence of our anatomical and symptom-association results highlights a structure–function relationship within these motor networks. Future studies are encouraged to expand on these findings by incorporating longitudinal data and multimodal imaging to track individualized disease progression and ultimately enable personalized, network-based diagnostics in PD.

## Data Availability

The raw data supporting the conclusion of this article will be made available by the authors upon reasonable request.
