# Supplementary material for: Volumetric signatures of basal ganglia–thalamo–cortical and cerebello–thalamo–cortical networks in Parkinson's disease and its motor subtypes
Source: Front Aging Neurosci. 2026 Feb 9;18:1743479. doi: 10.3389/fnagi.2026.1743479 (PMC12926348; doi:10.3389/fnagi.2026.1743479)
Supplement: Supplementary file 1 [file Data_Sheet_1.docx]

Supplementary Material

# Supplementary Figures


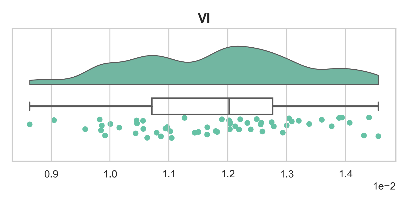

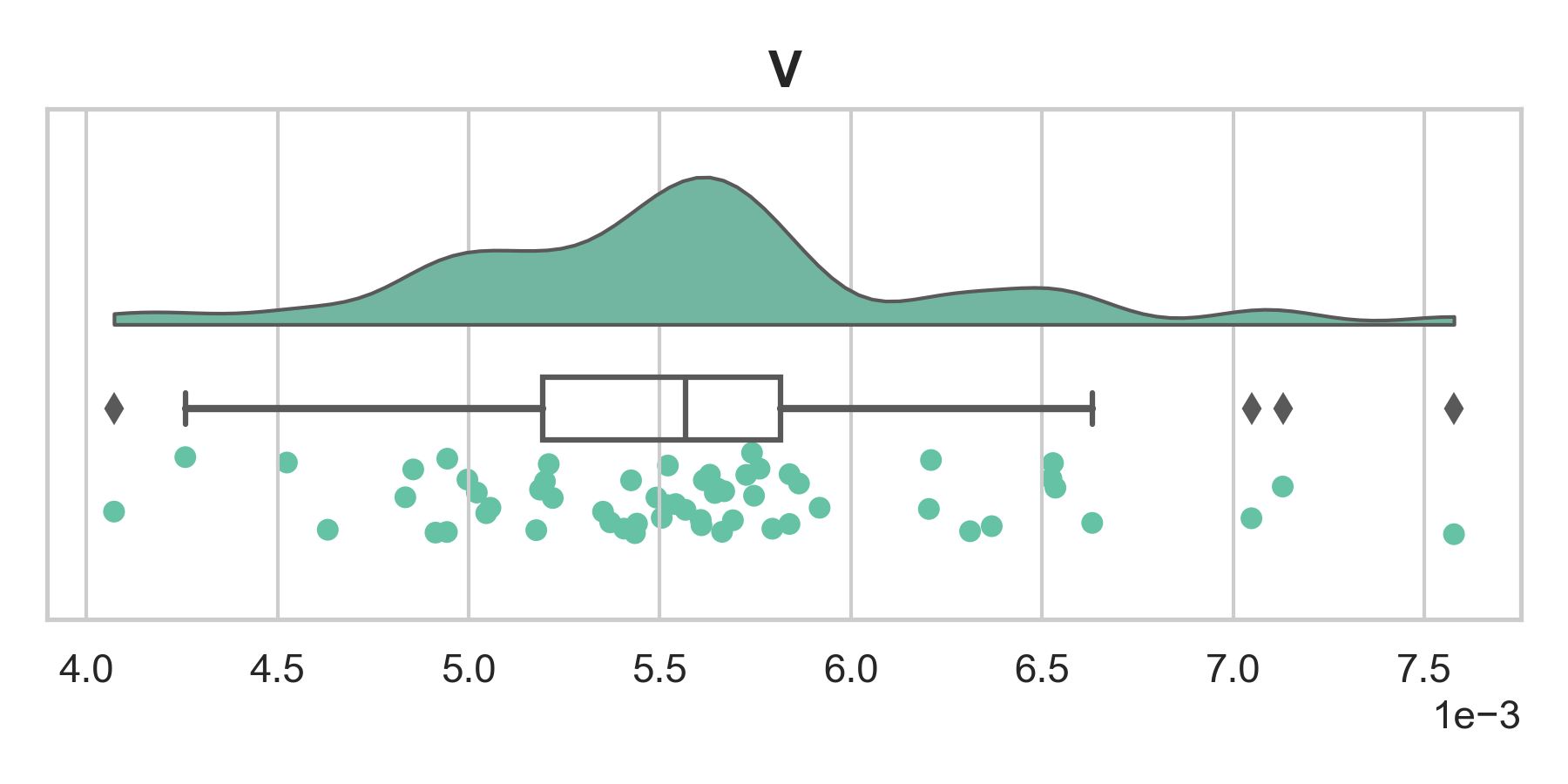

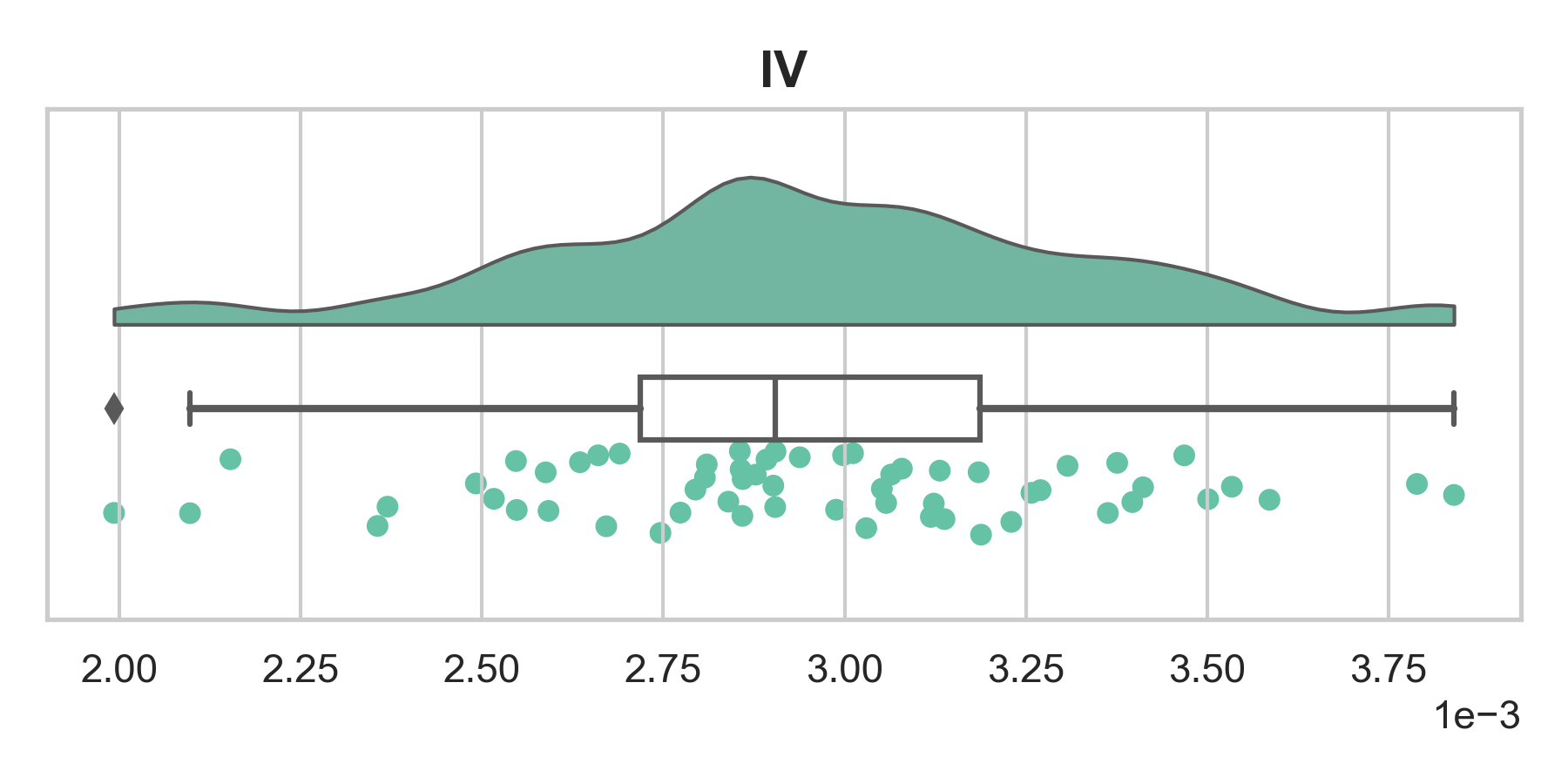

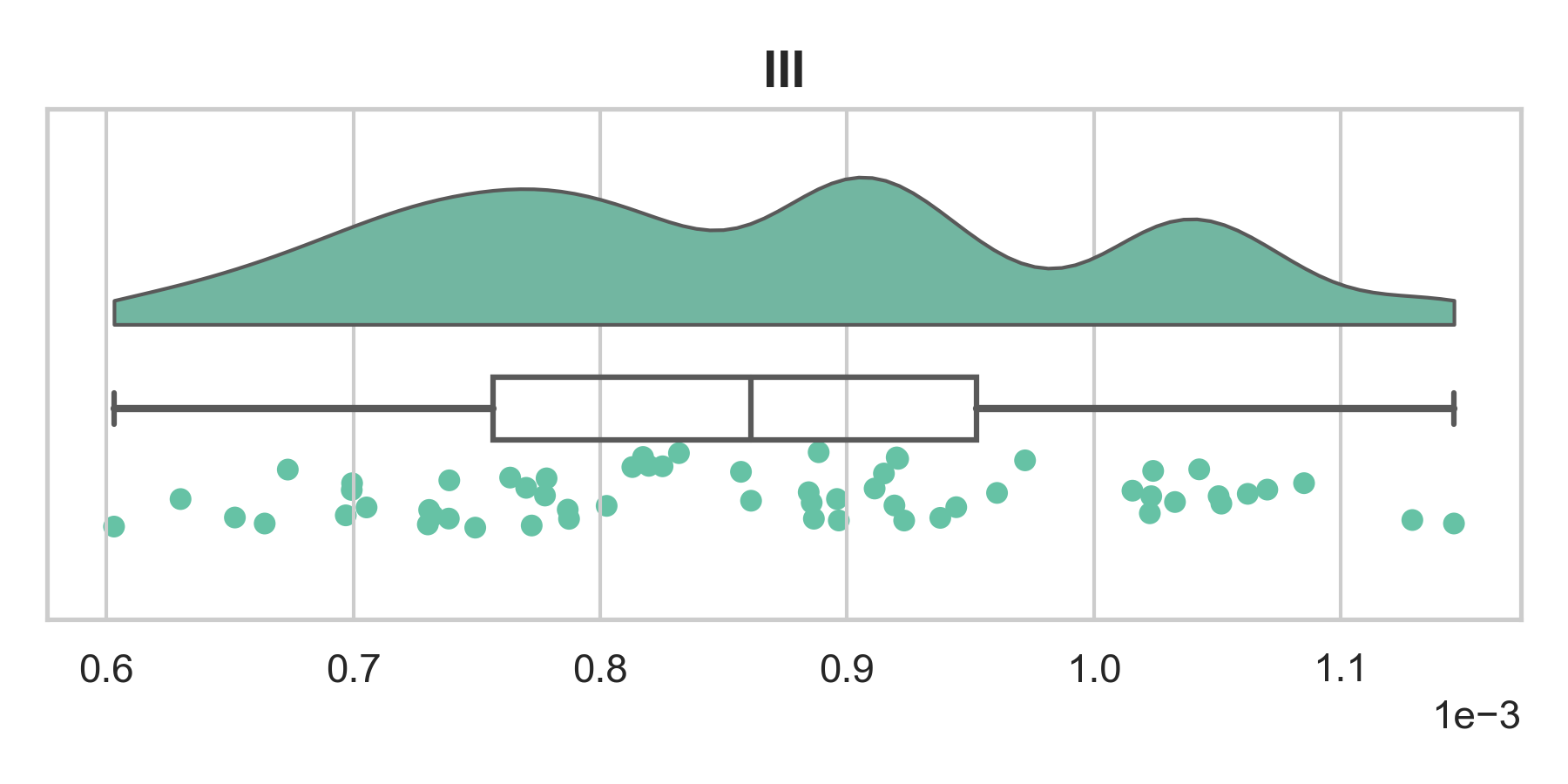

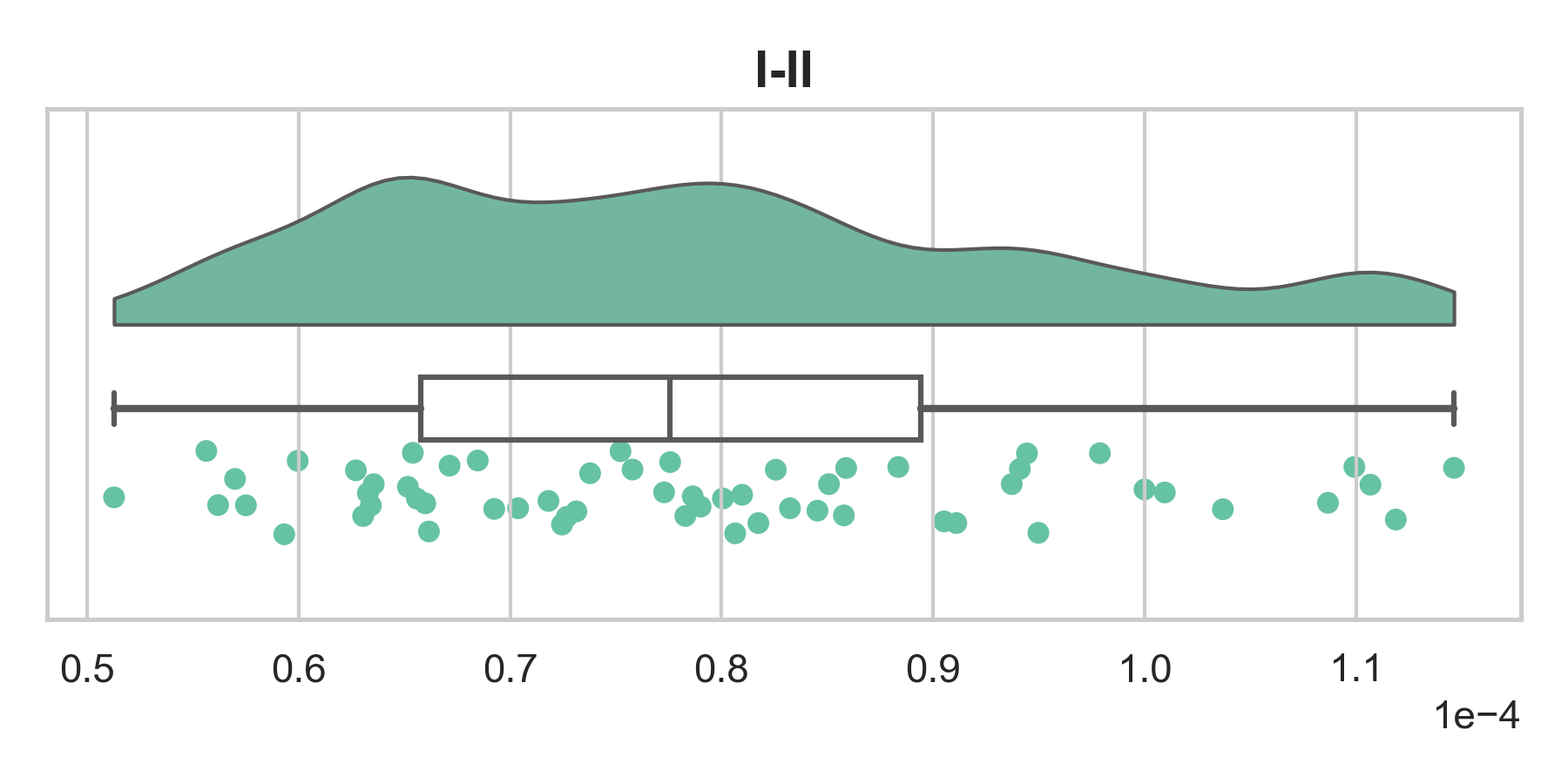

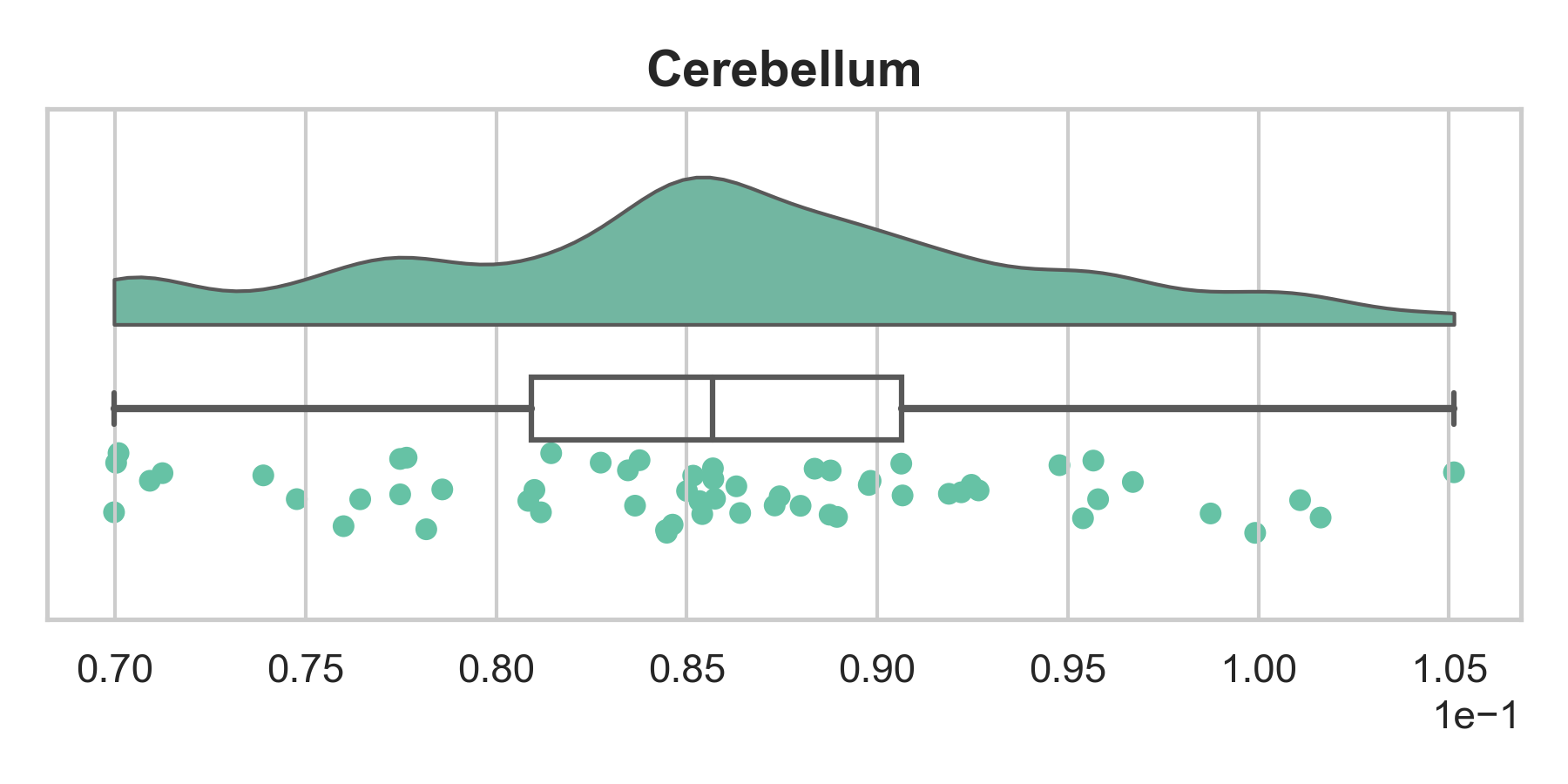

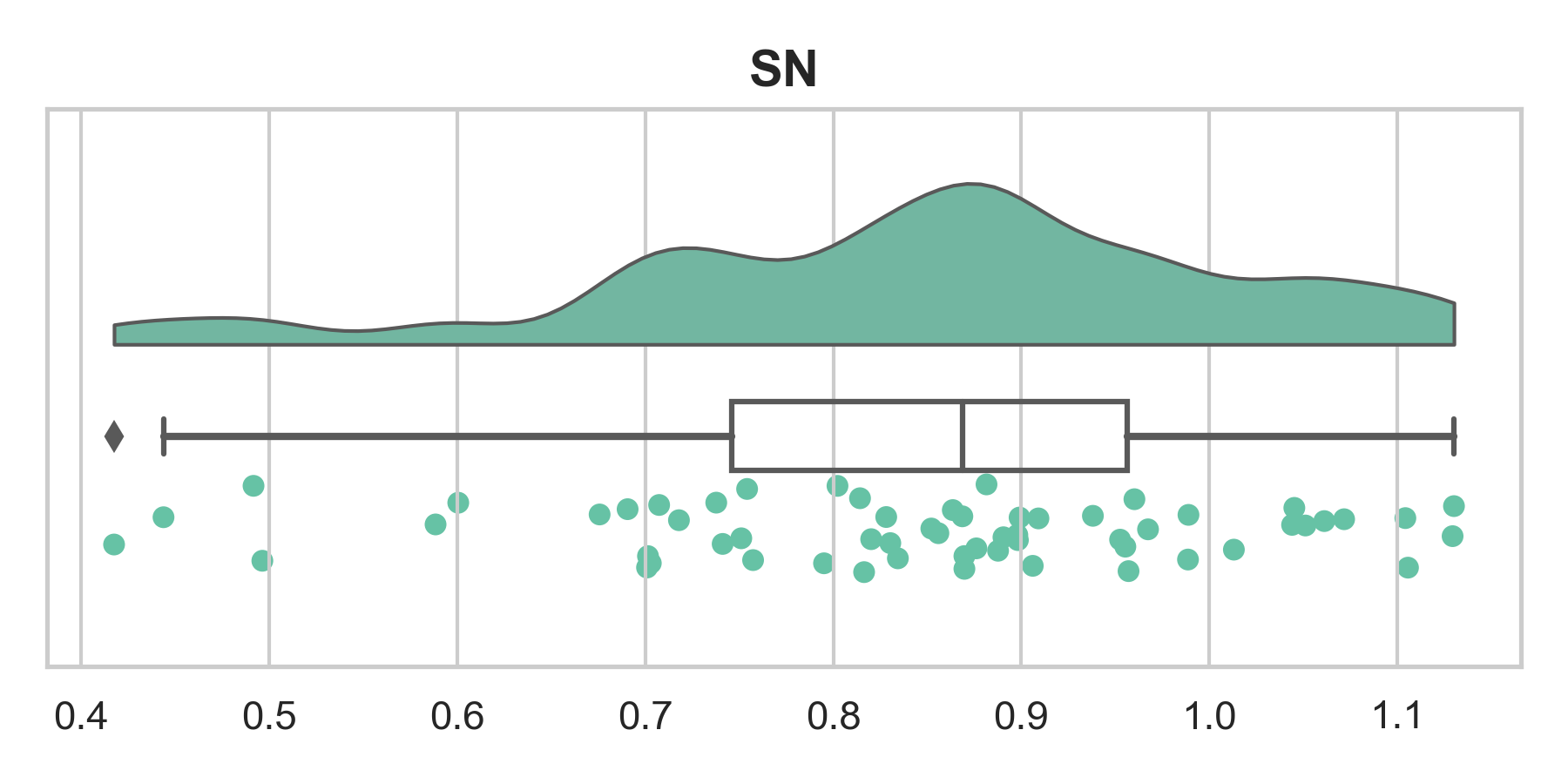

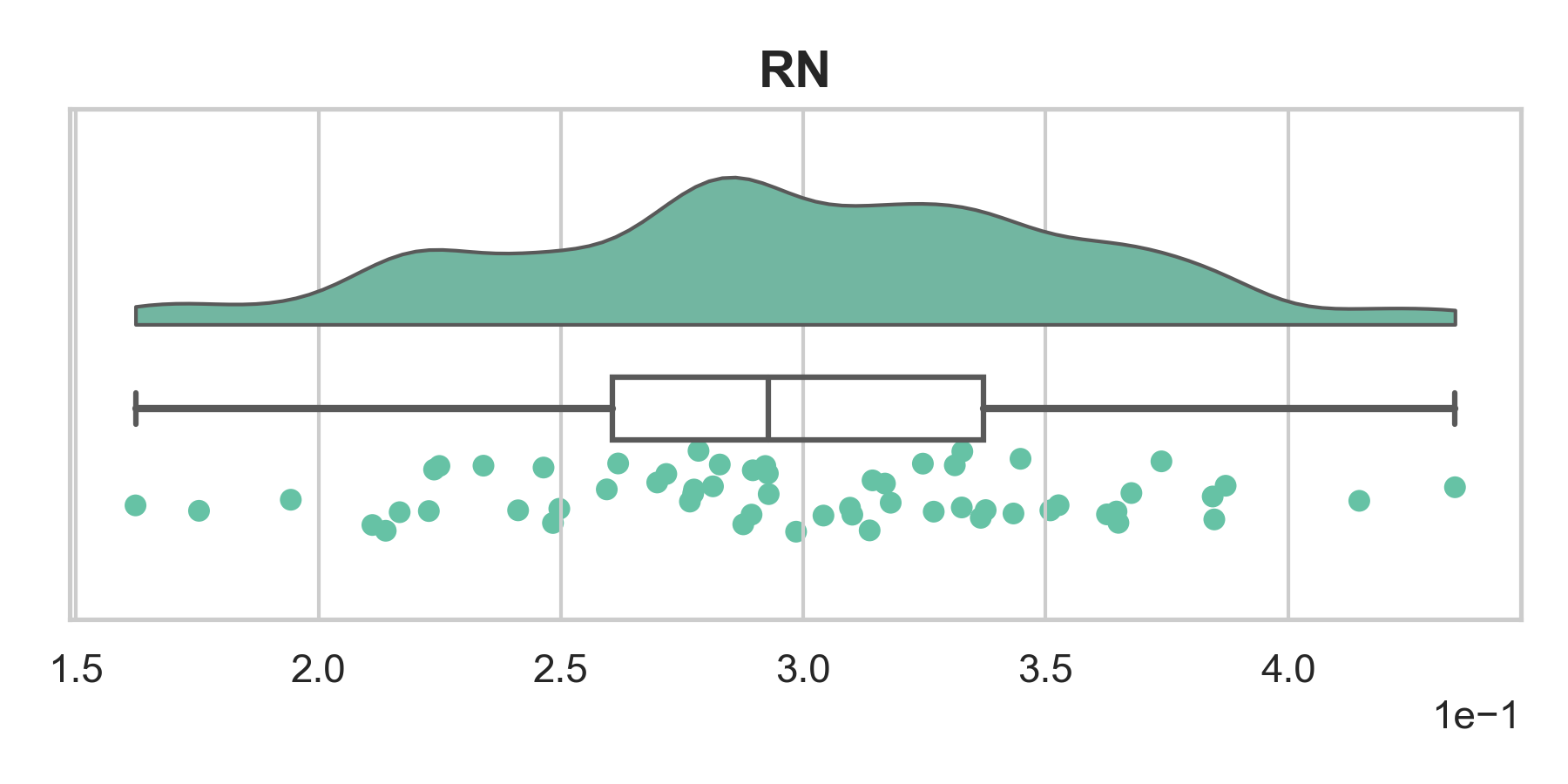

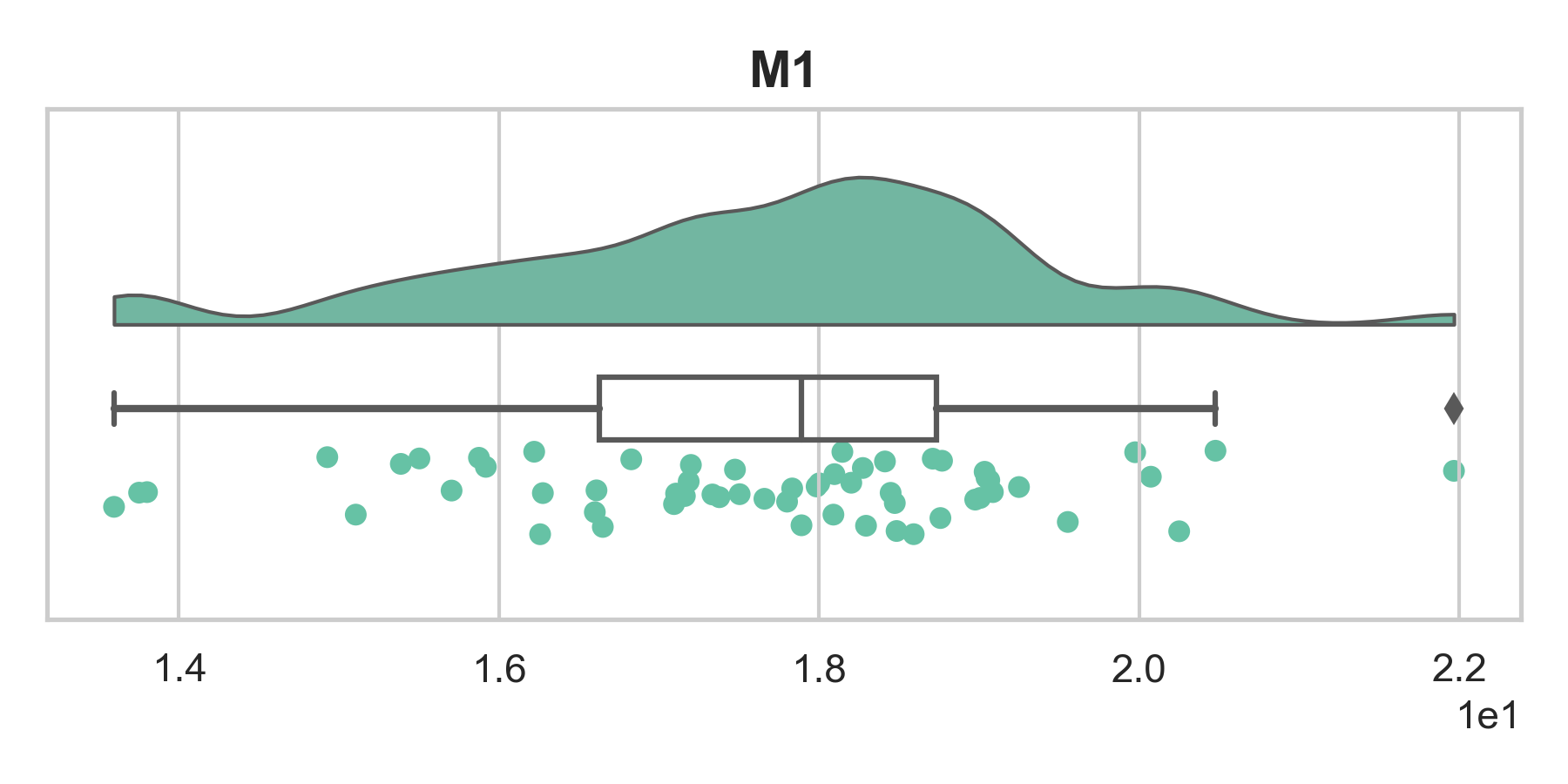

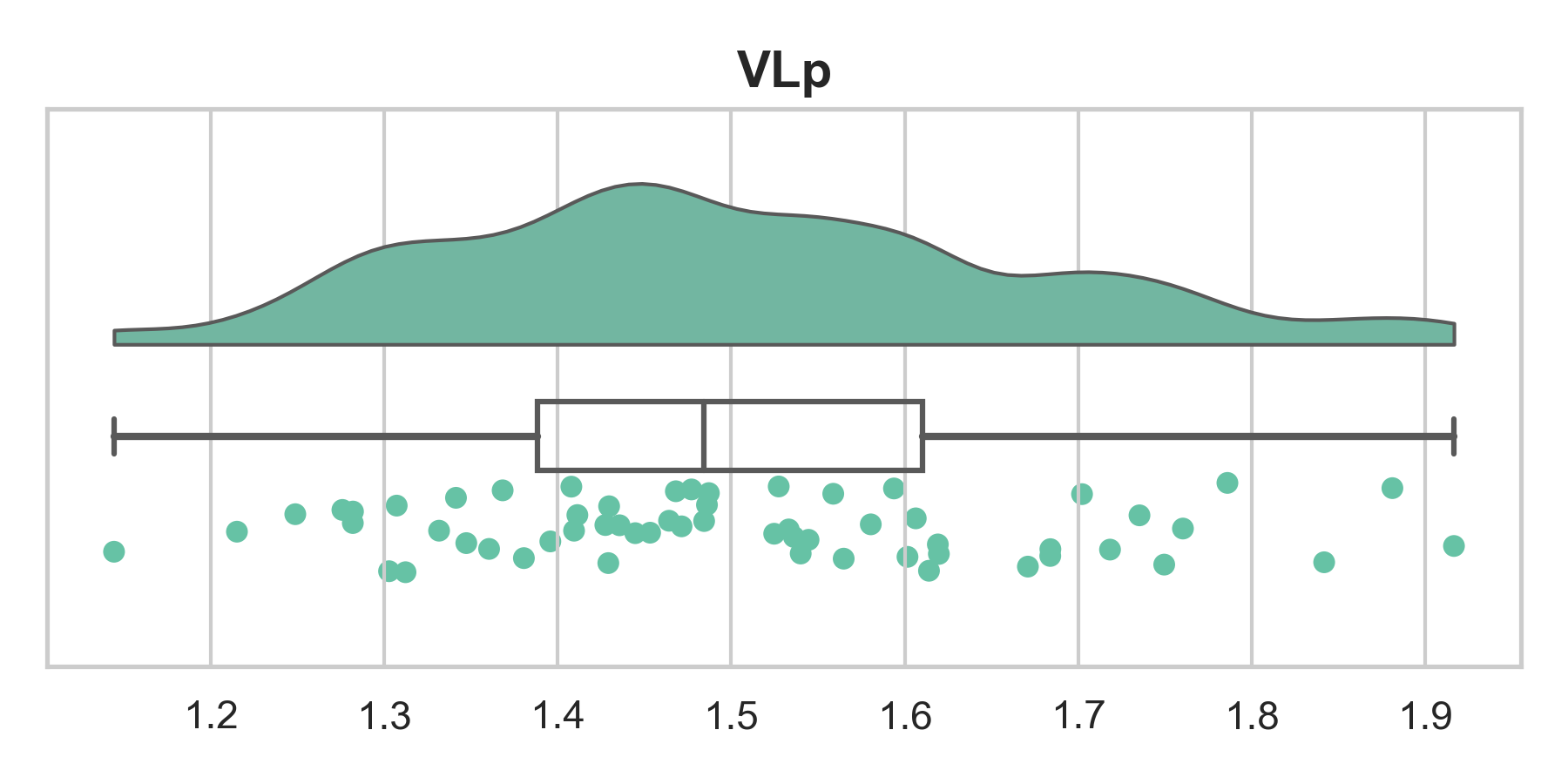

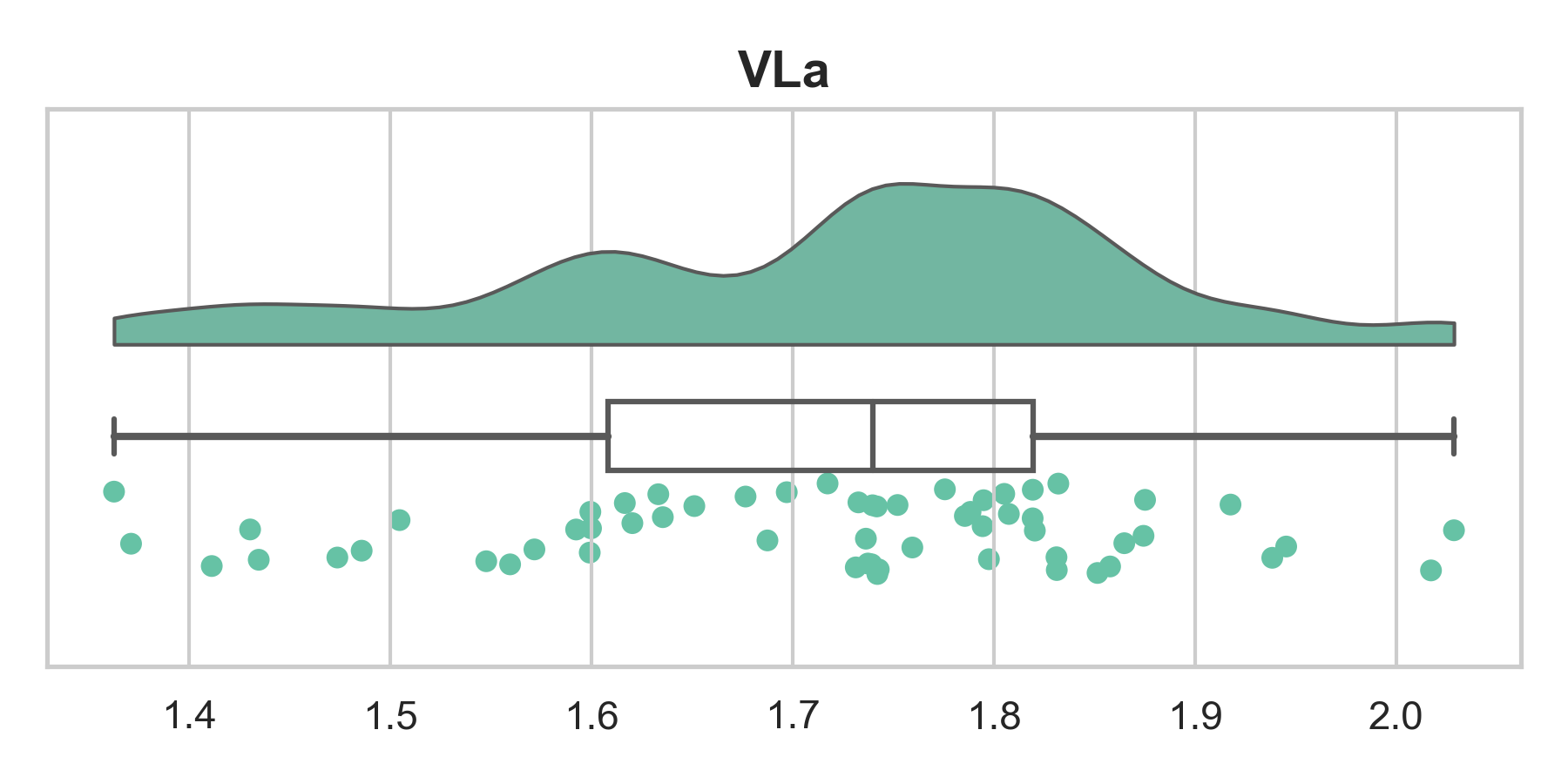

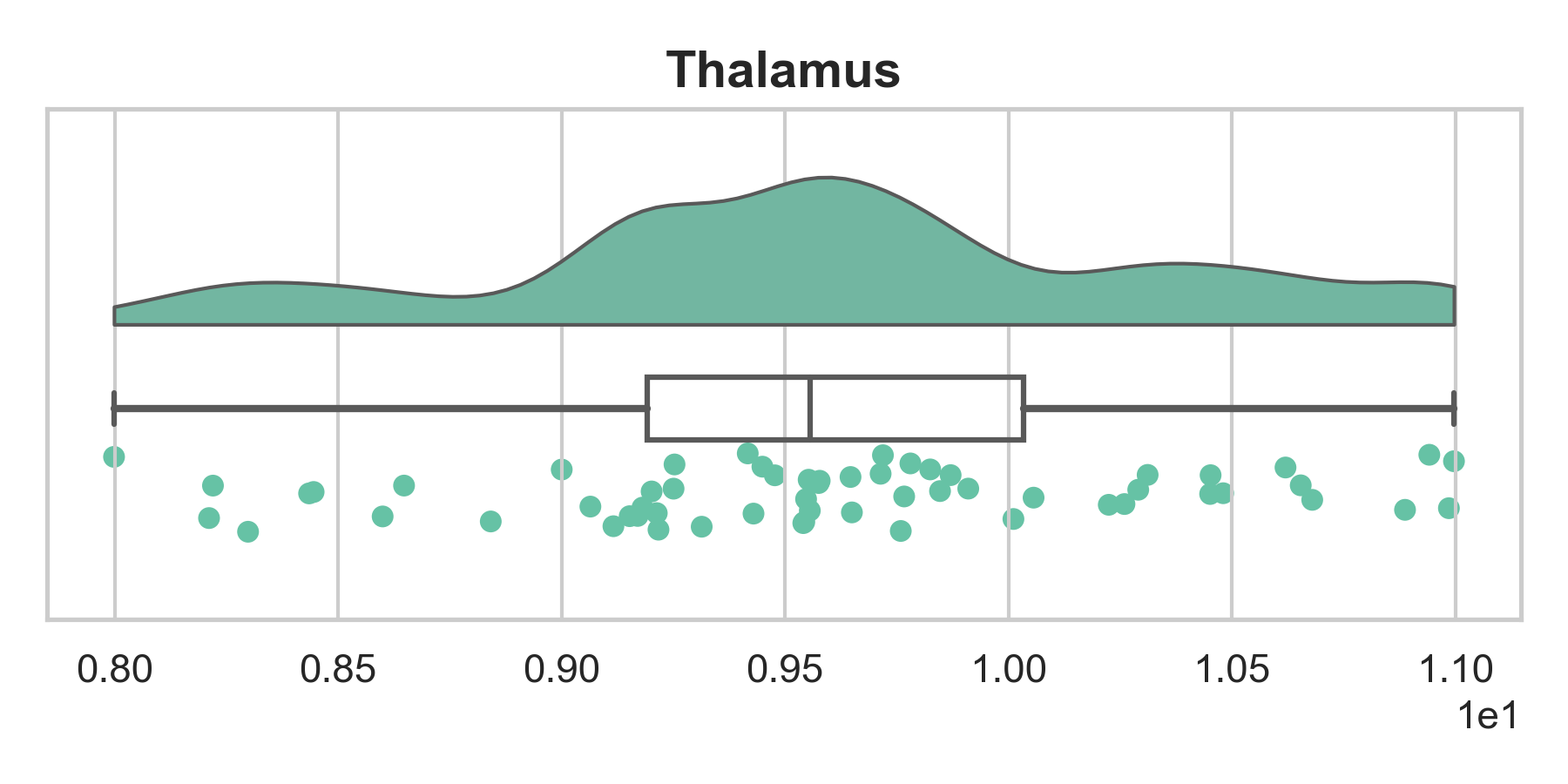

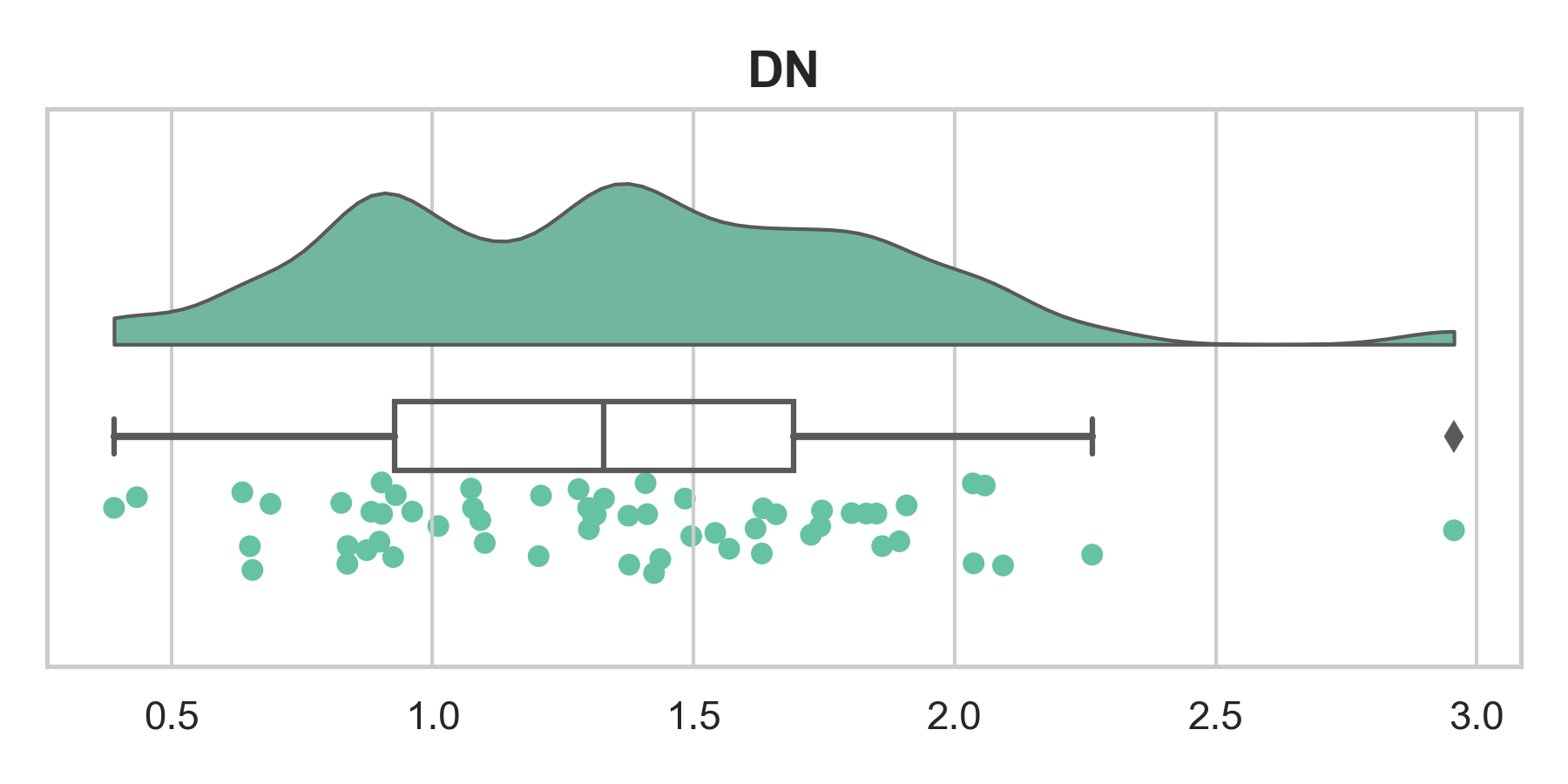

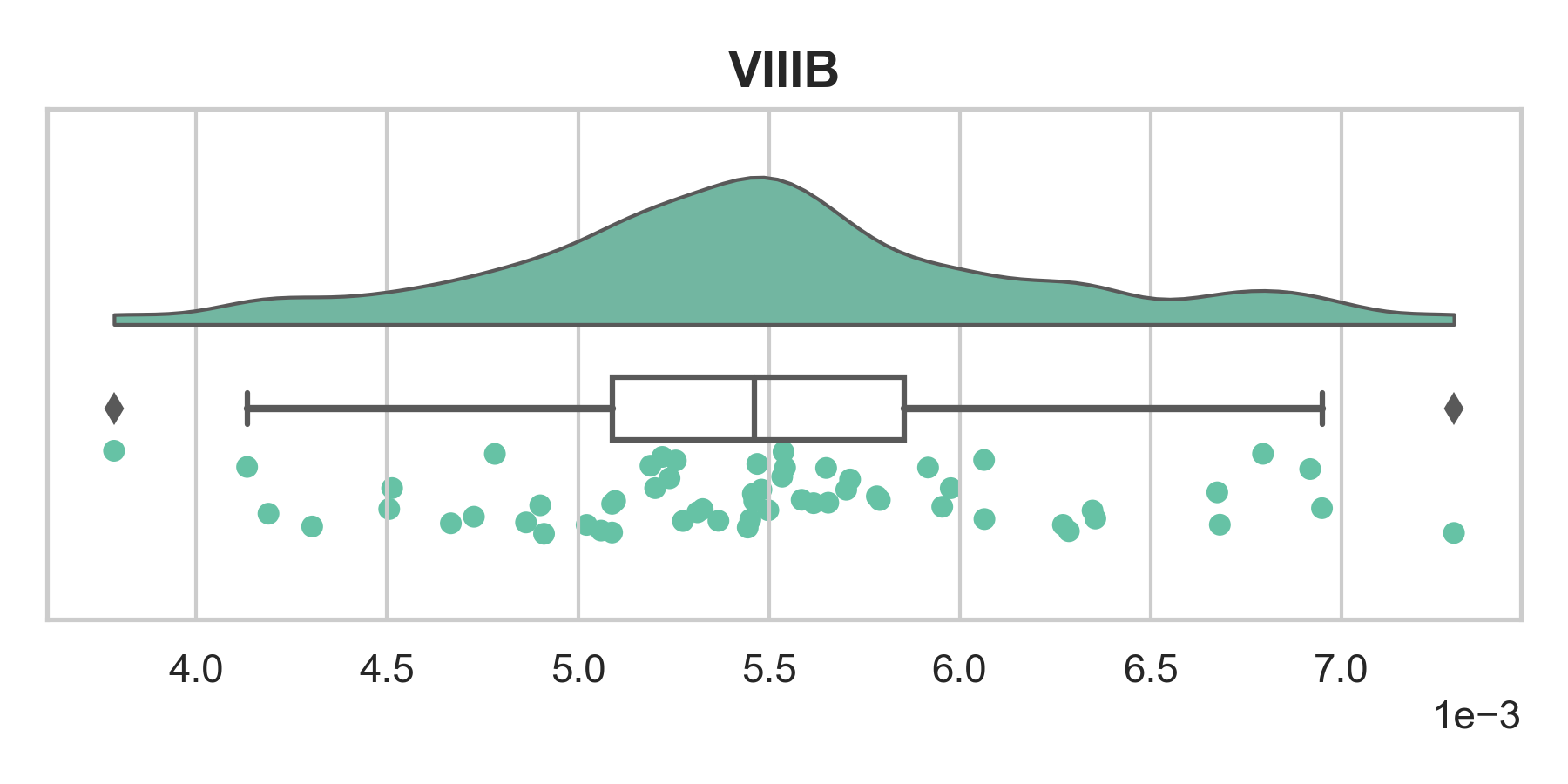

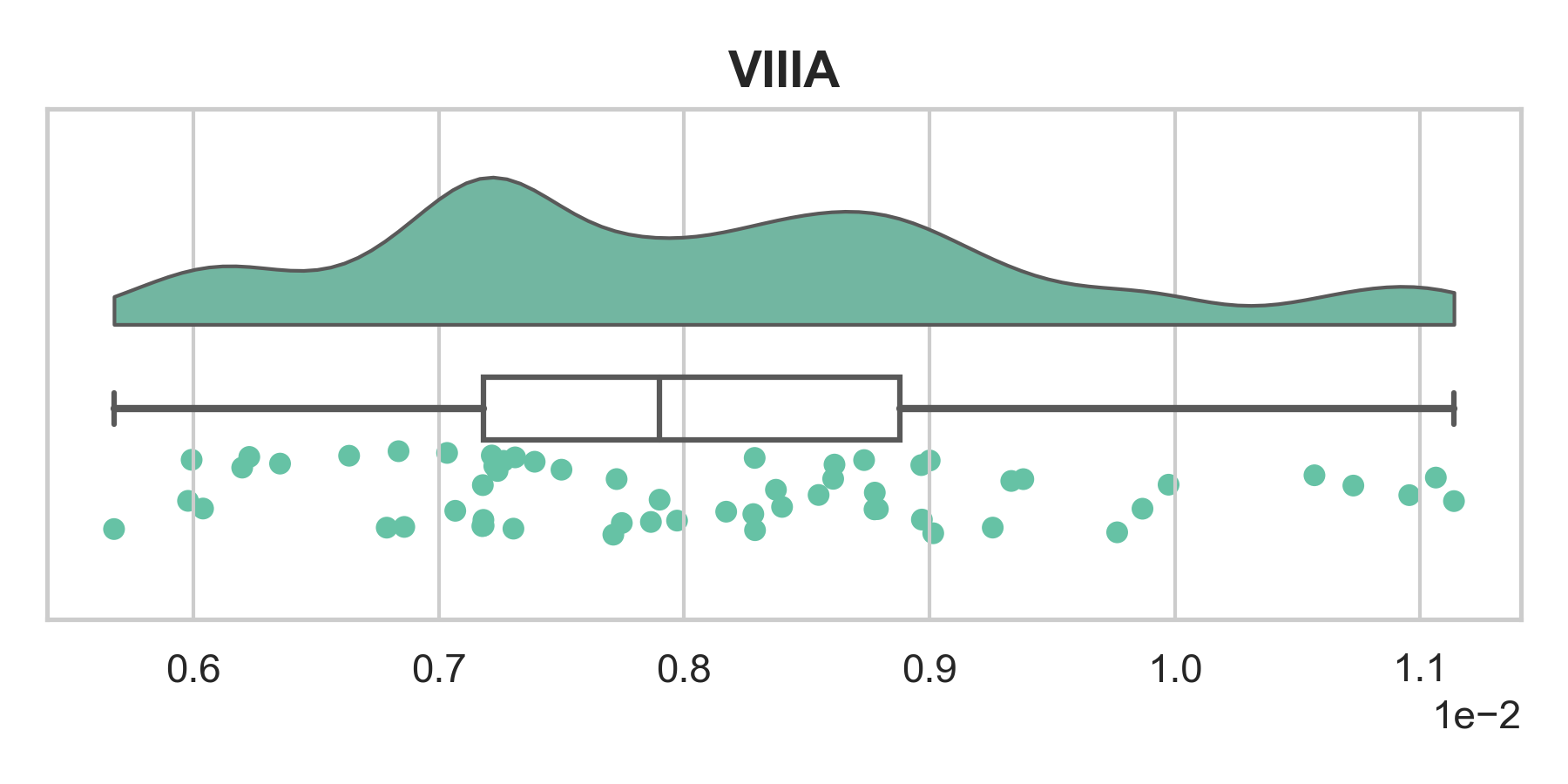

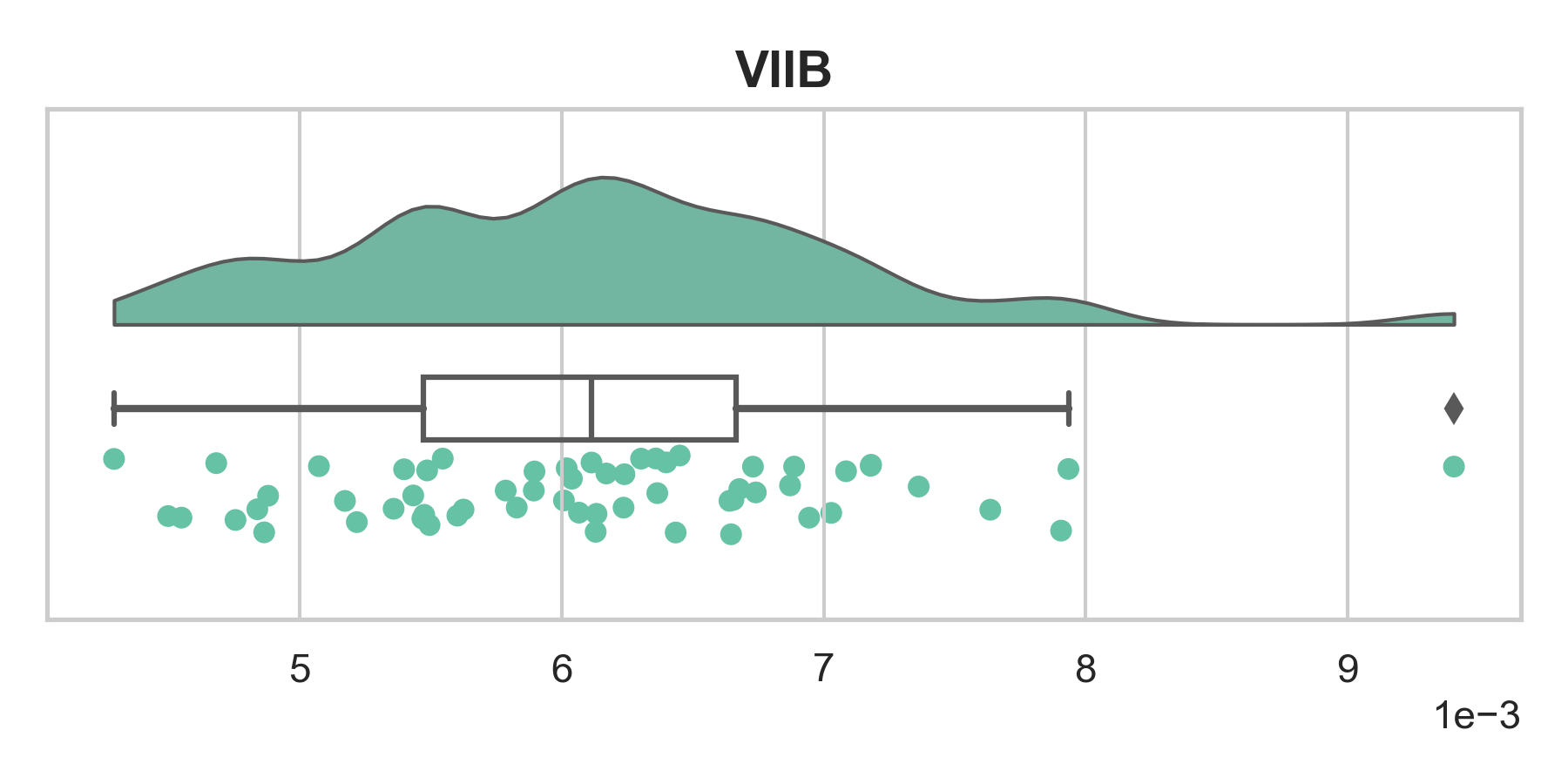

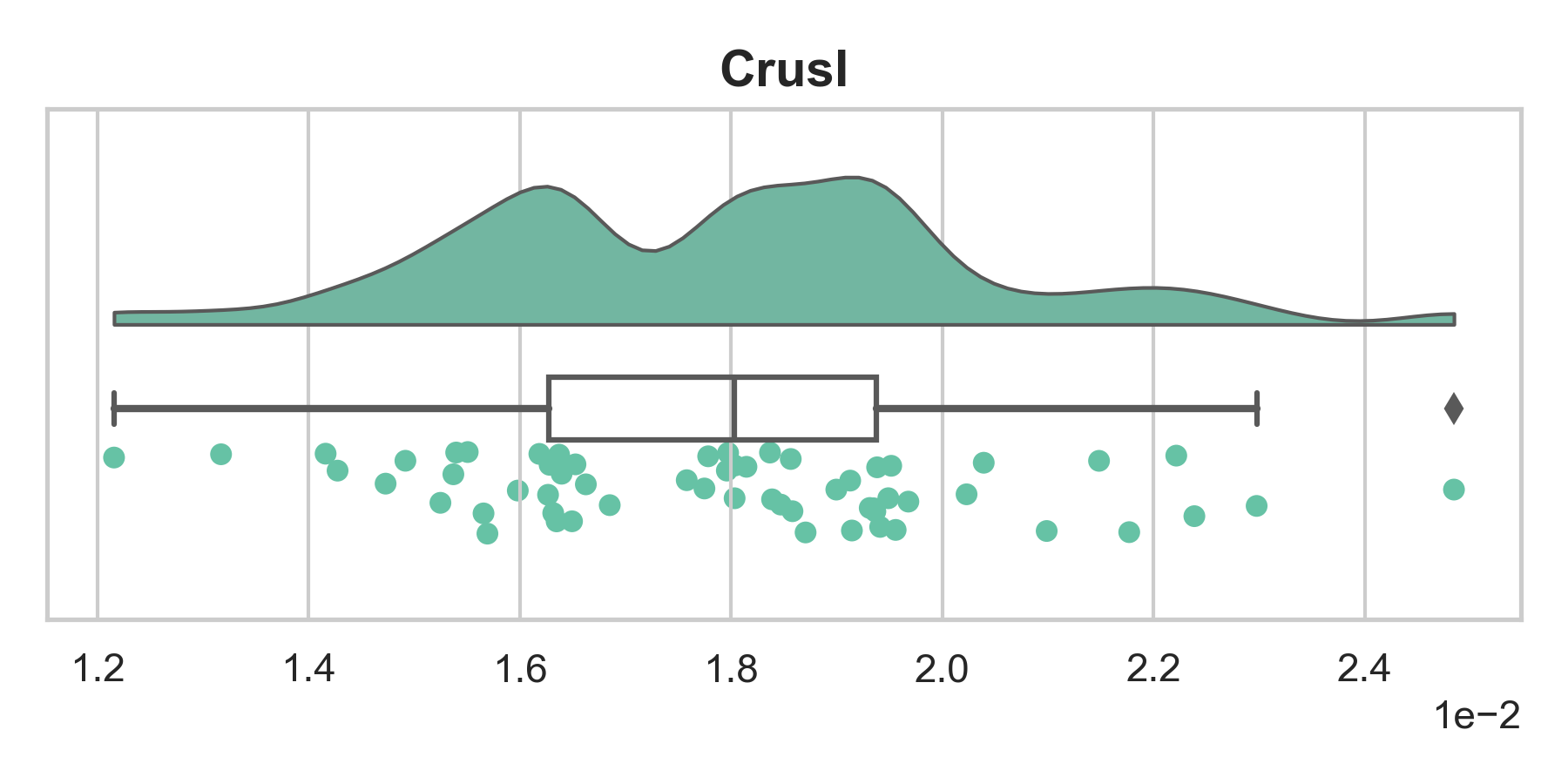

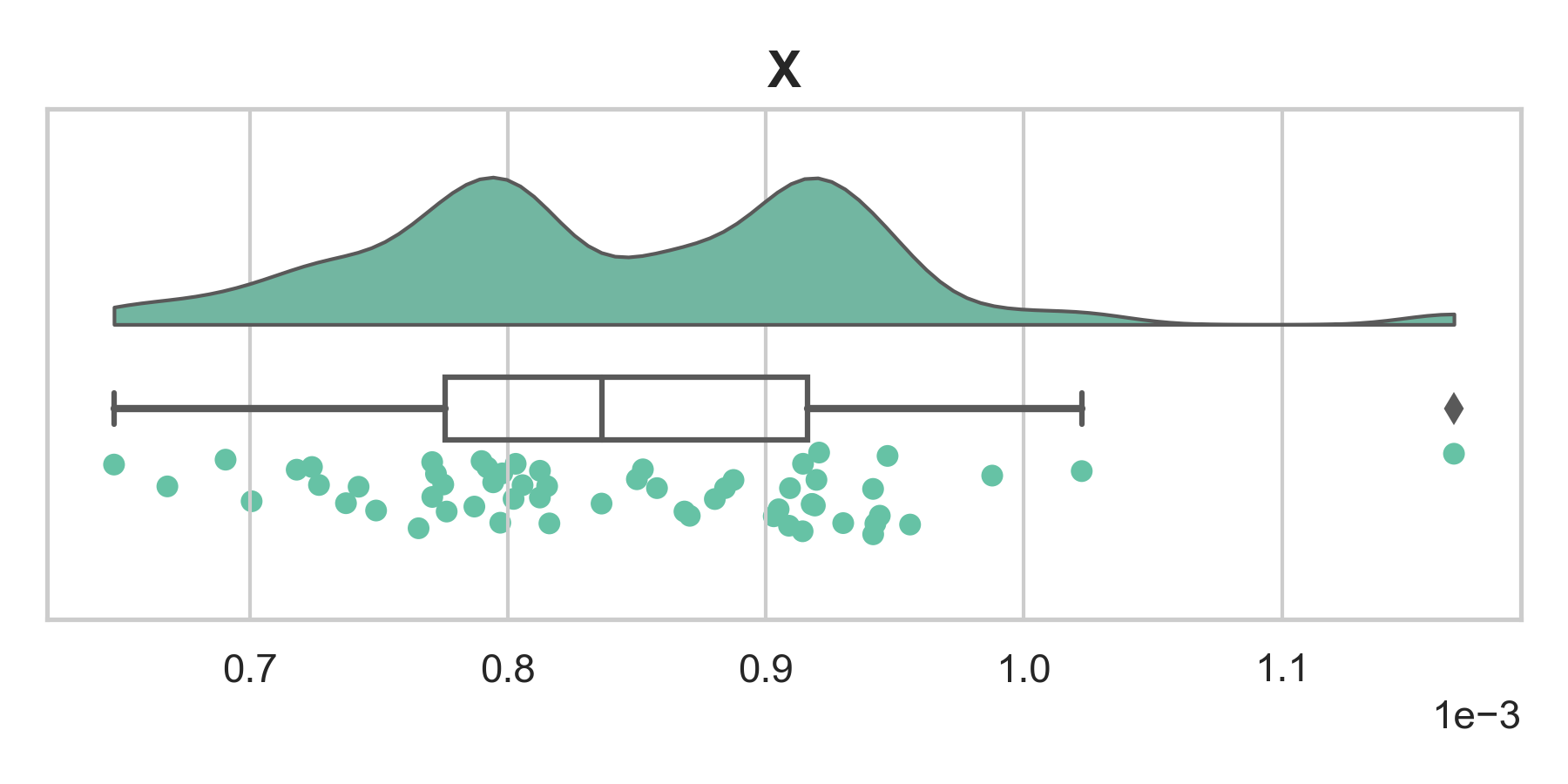

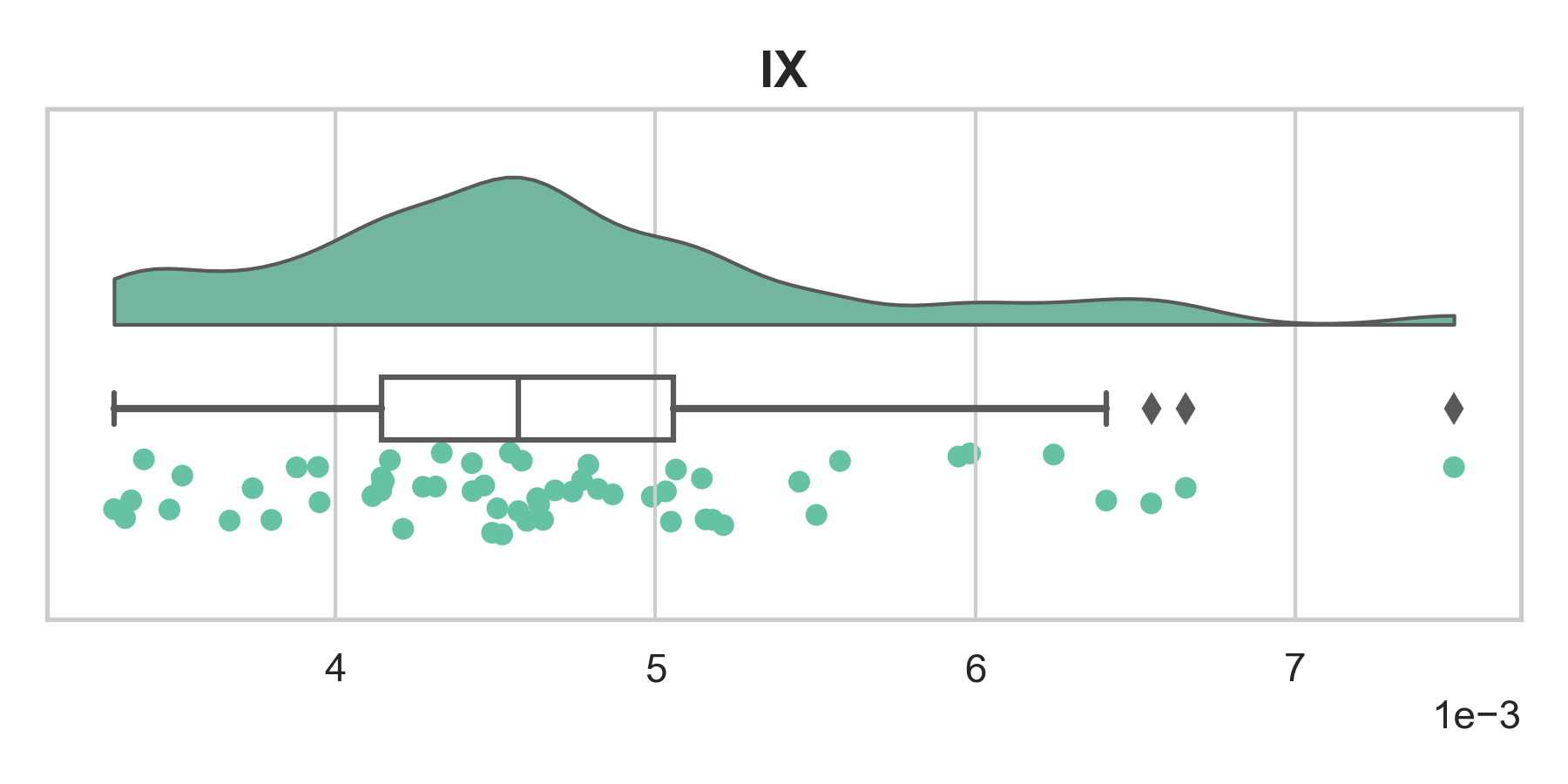

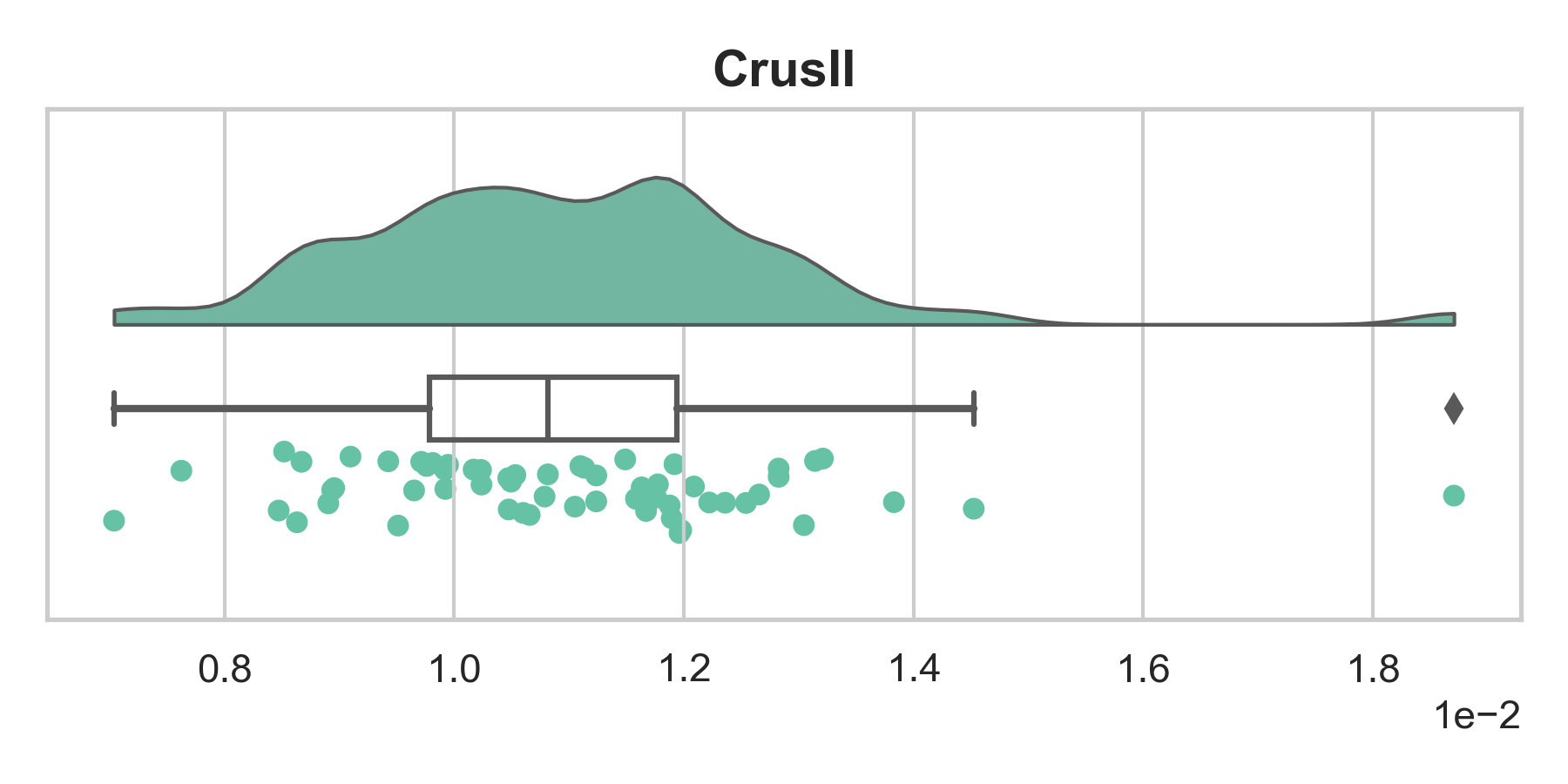


Supplementary Figure S1. Raincloud plots illustrating the distribution of normalized regional brain volumes across all participants. Each plot combines a density curve, boxplot, and individual data points to visualize the spread, central tendency, and potential outliers for each region of interest (ROI). Shown are volumes from key motor-related structures such as the dentate nucleus (DN), red nucleus (RN), ventral lateral posterior (VLp) and anterior (VLa) thalamic nuclei, substantia nigra–subthalamic complex (SN–STN or SN), and cerebellar lobules I–X. Notably, volumes were homogeneously distributed and exhibited near-normal patterns across groups, supporting the use of parametric modeling in subsequent analyses.


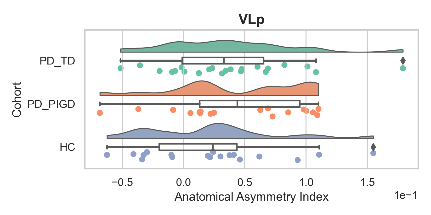

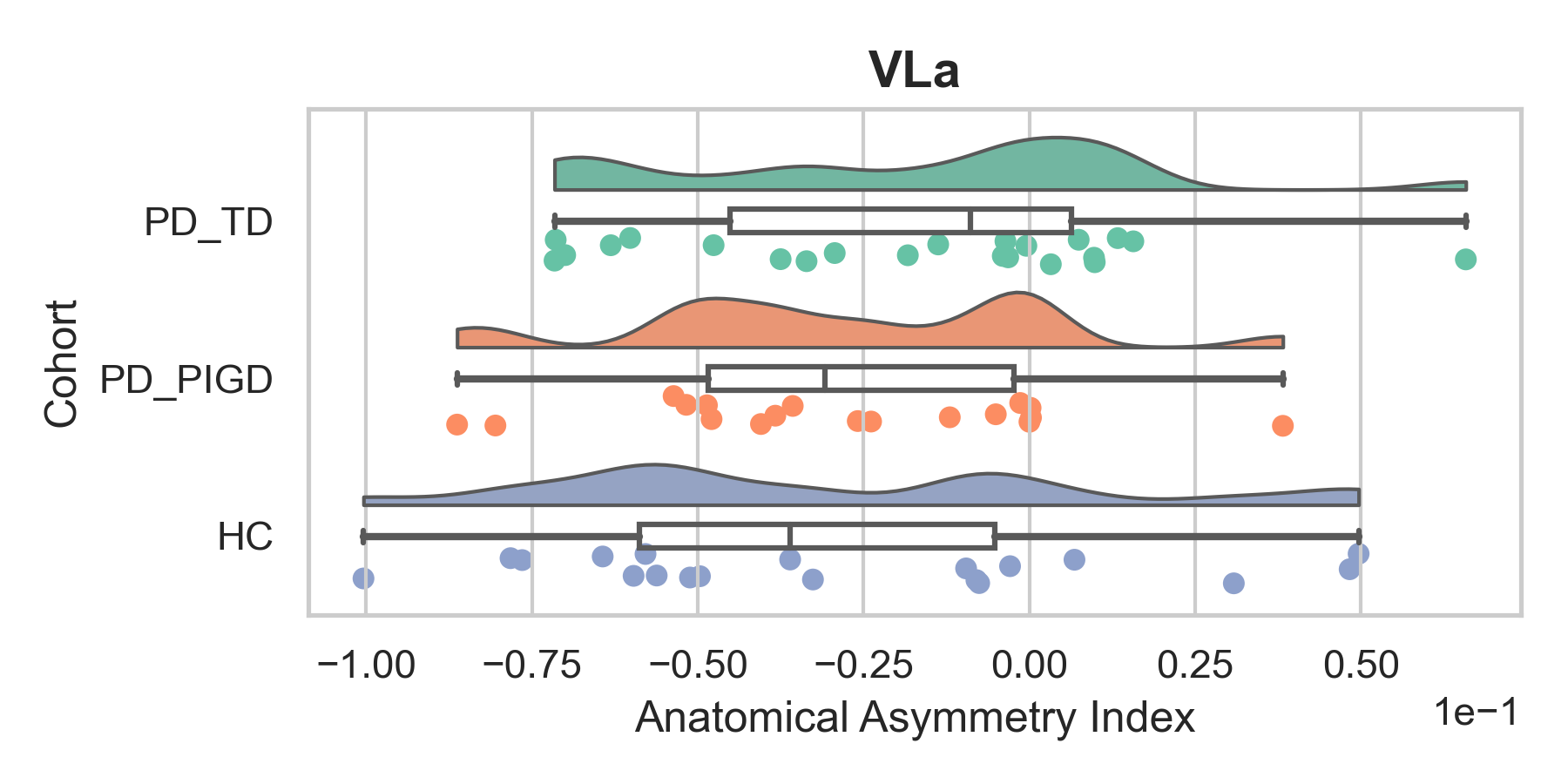

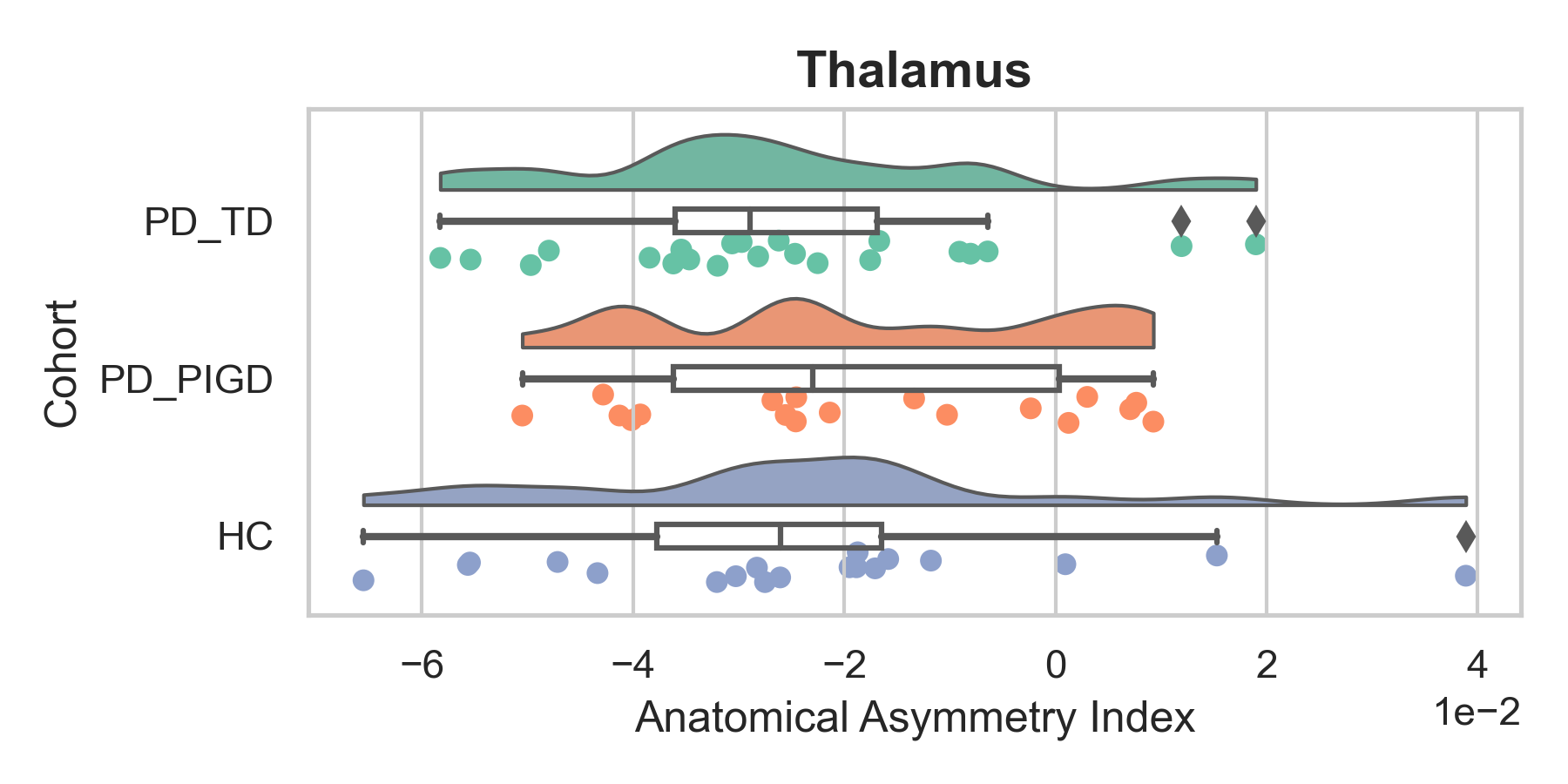

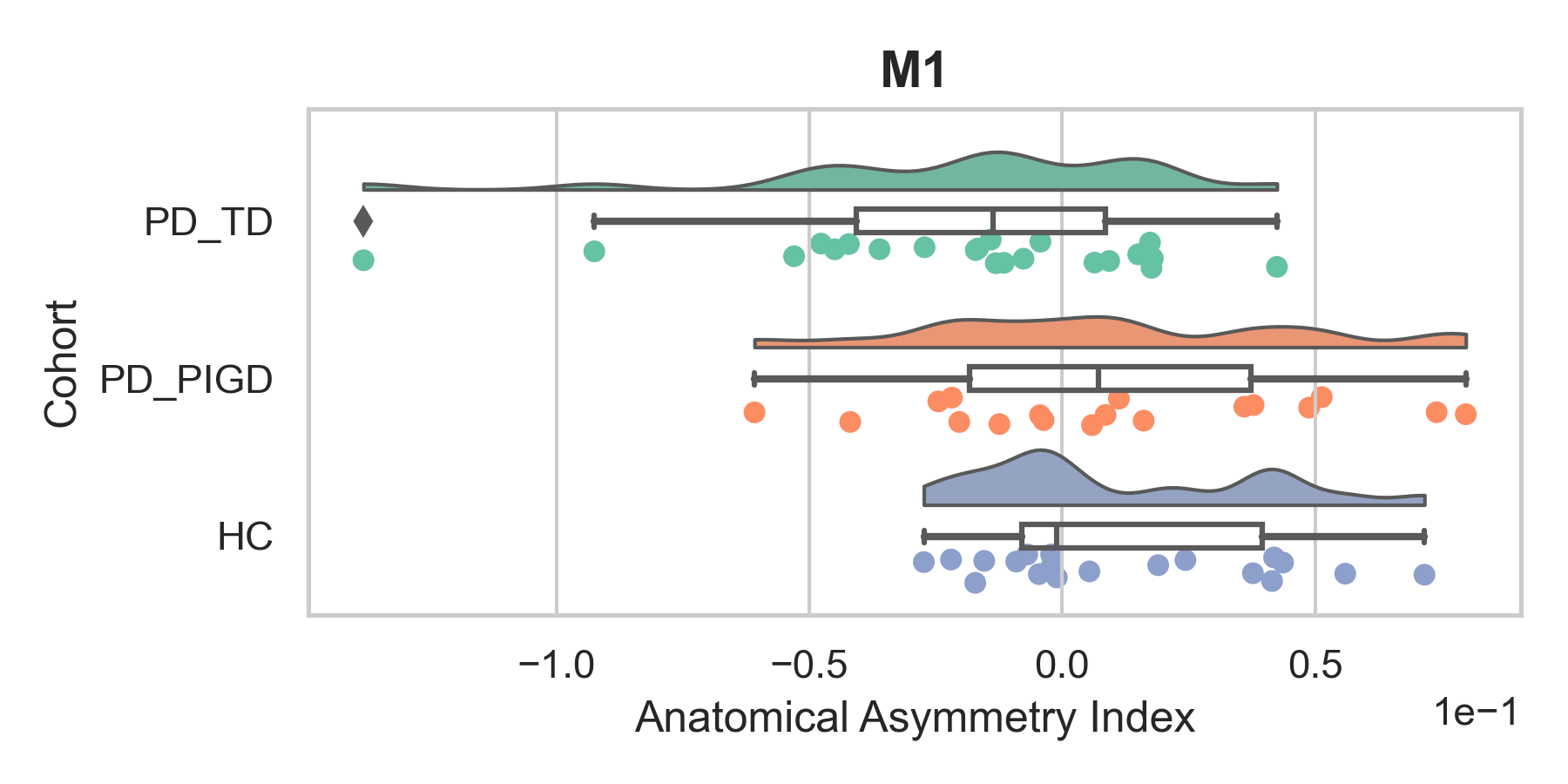

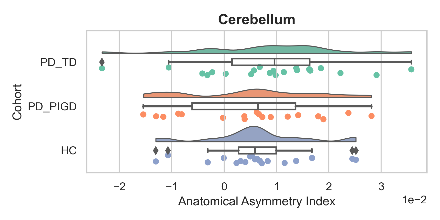

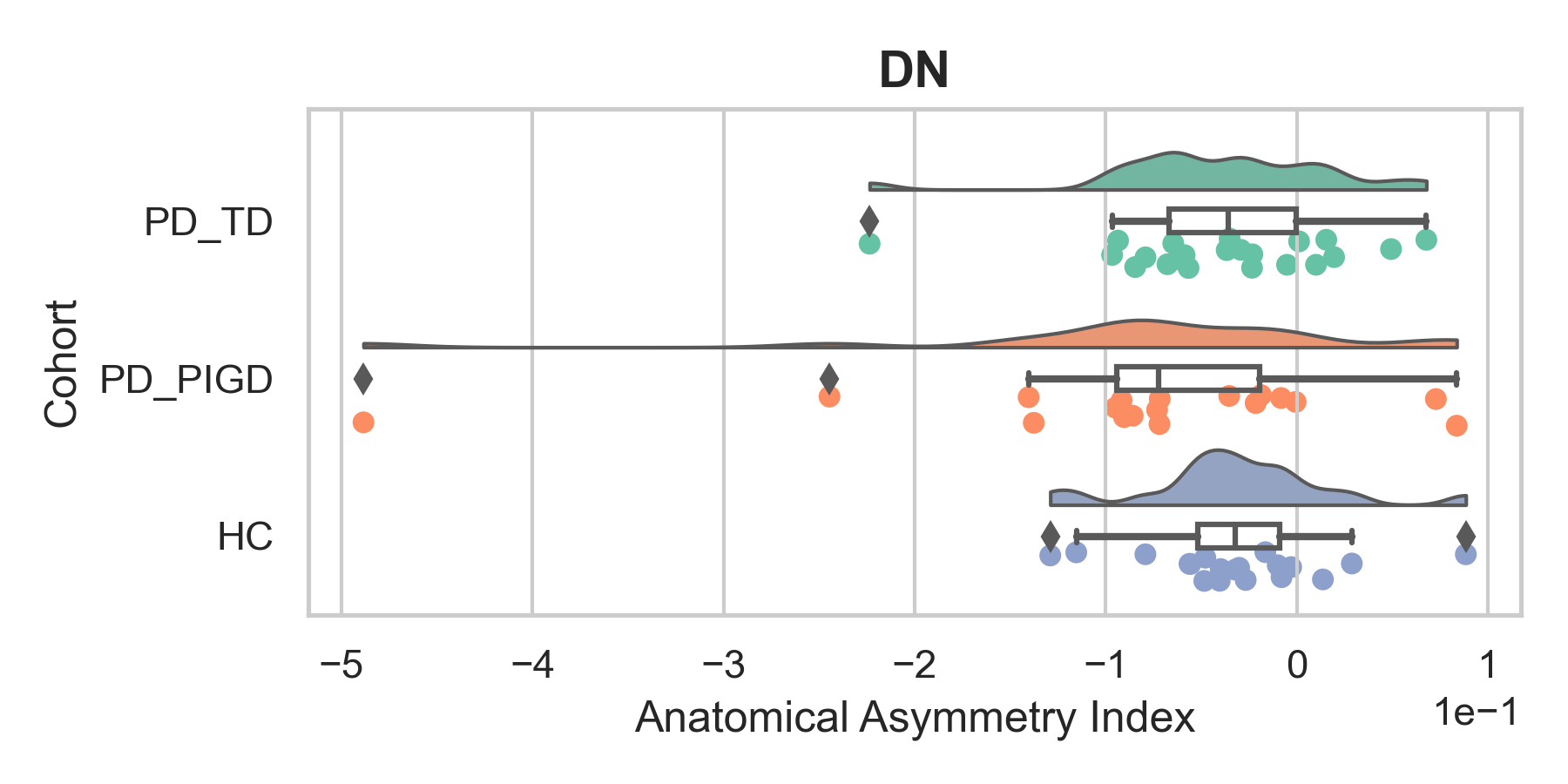

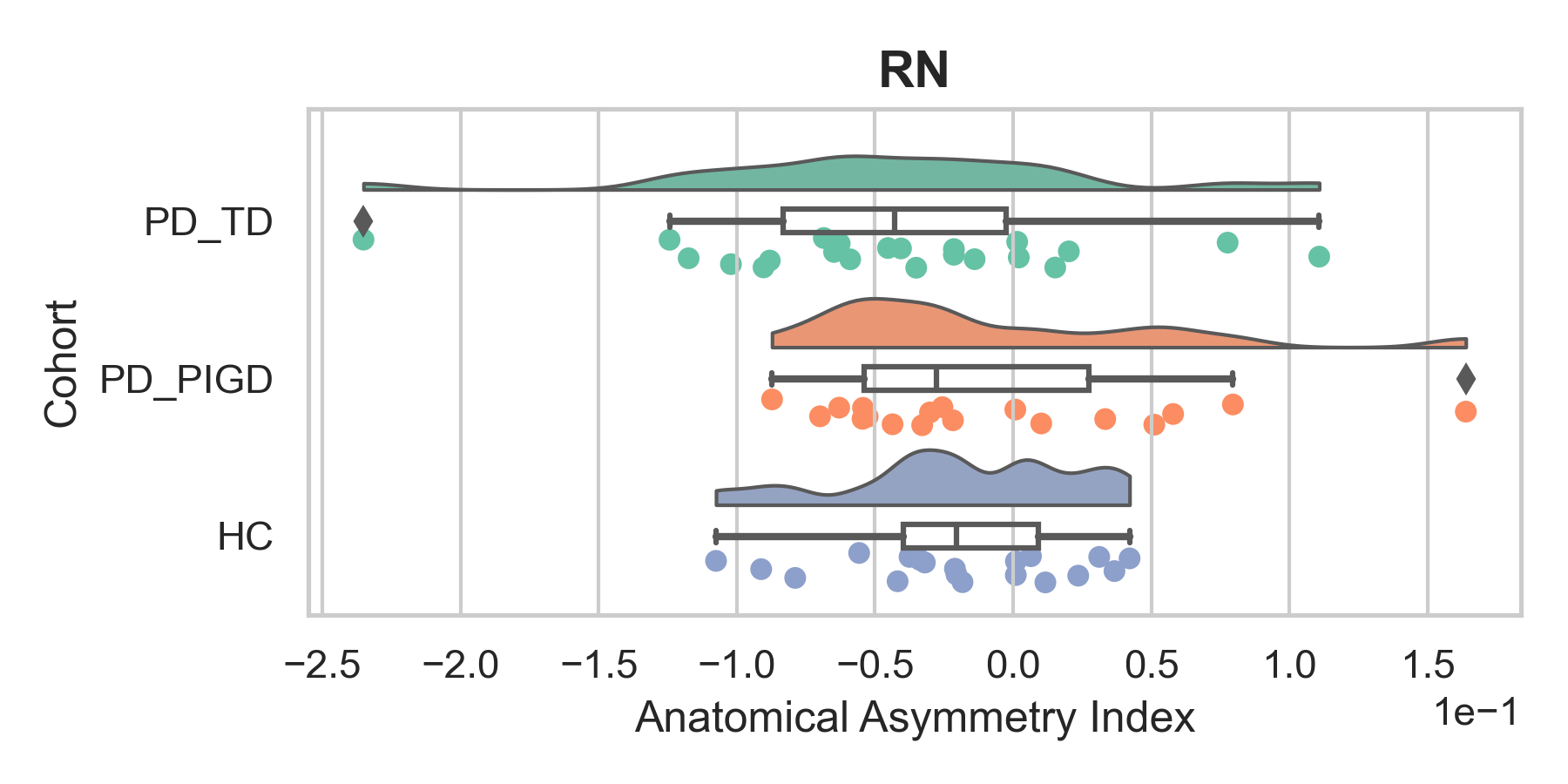

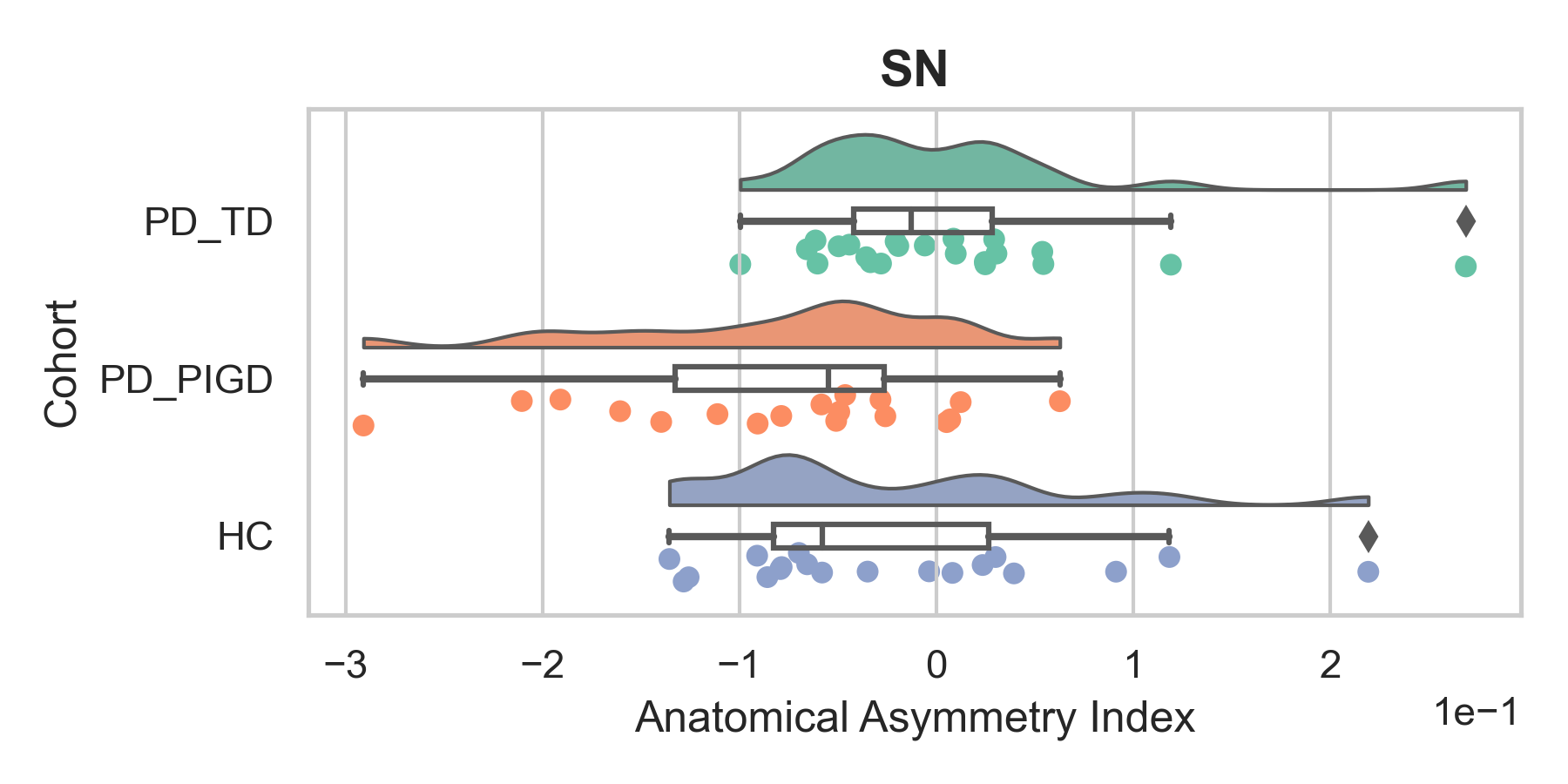

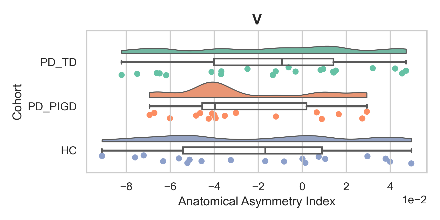

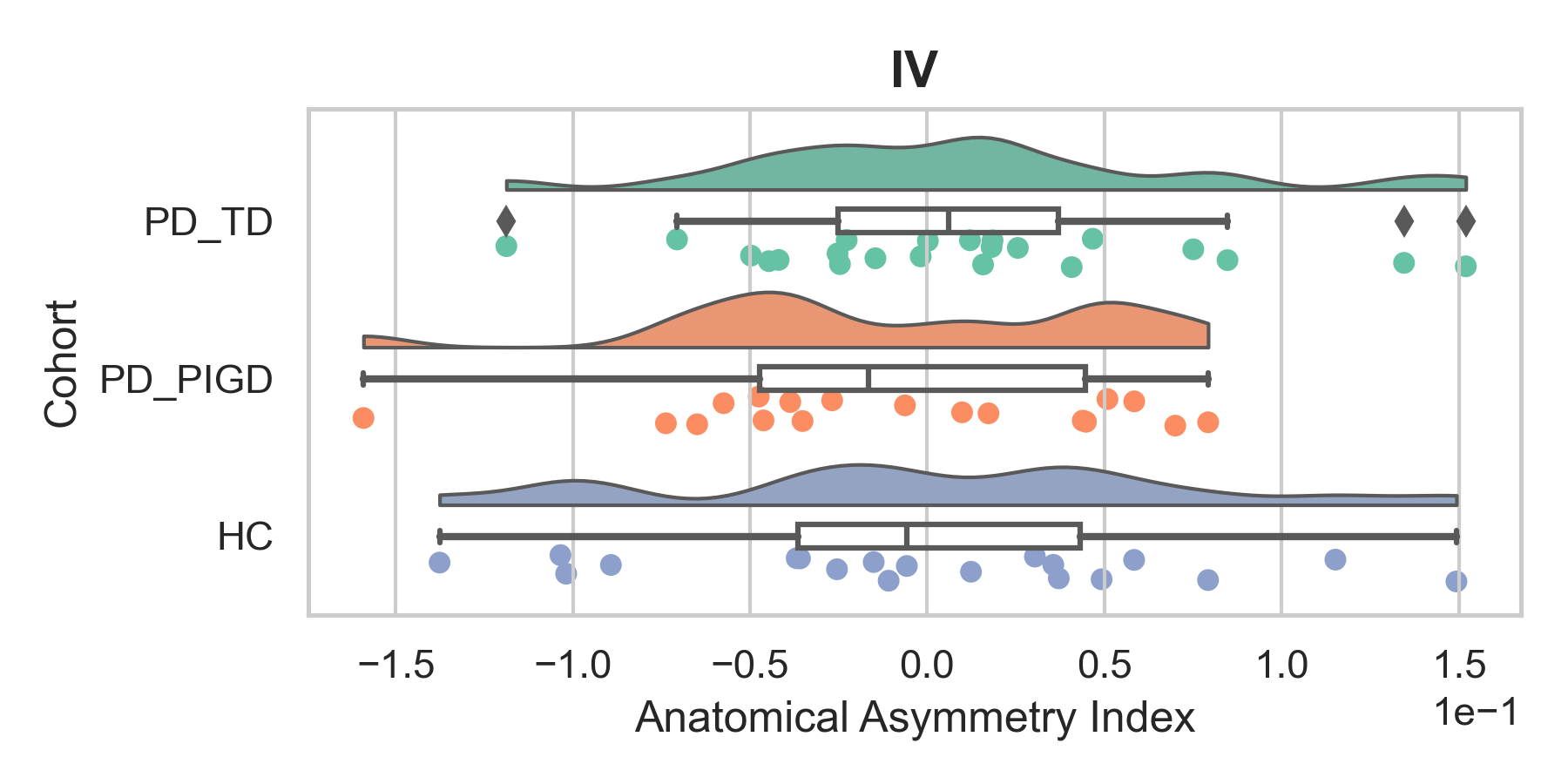

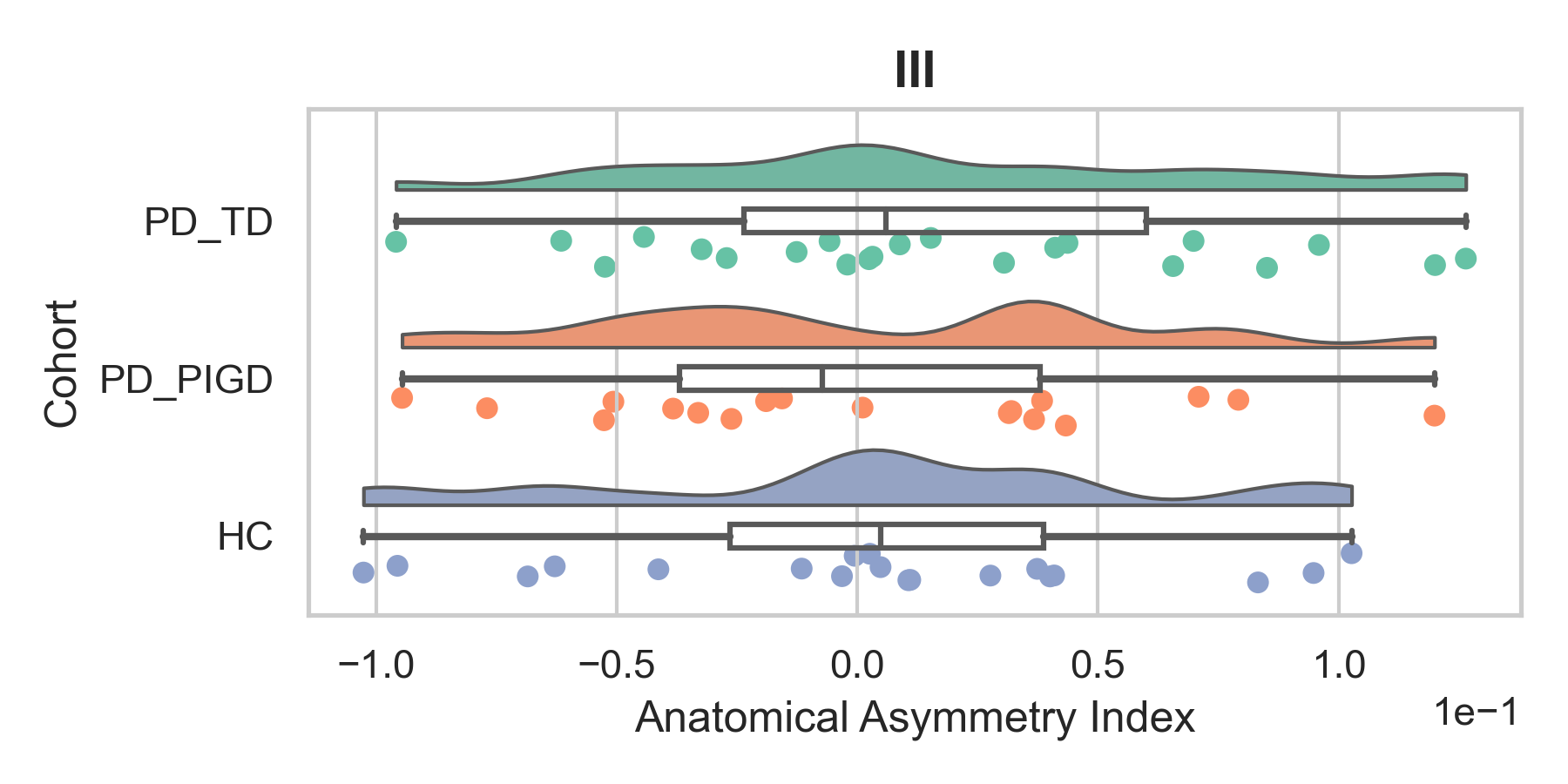

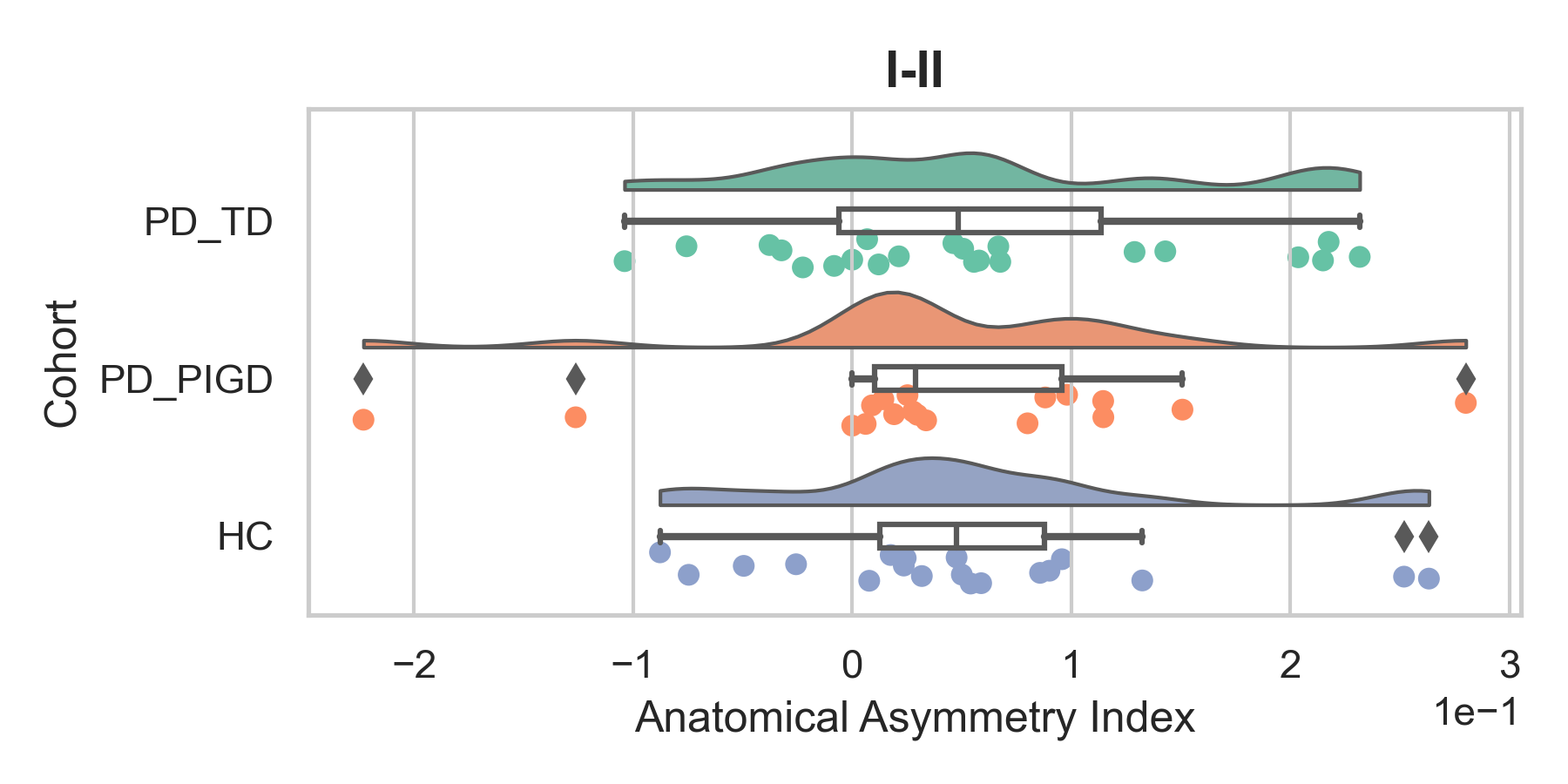

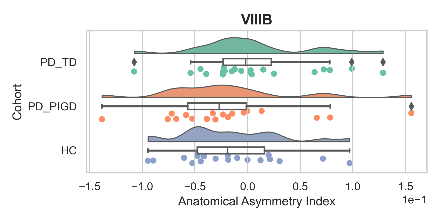

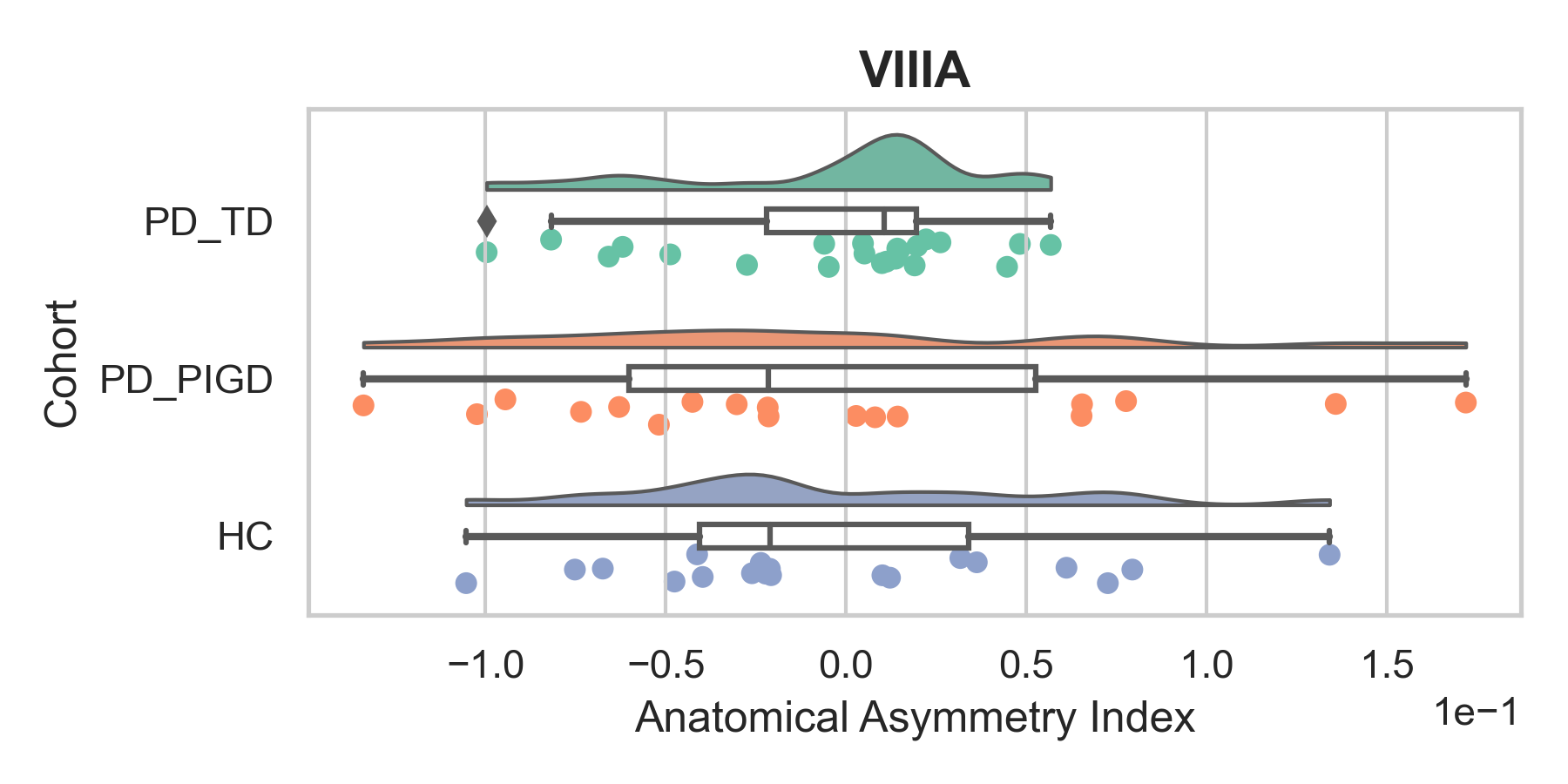

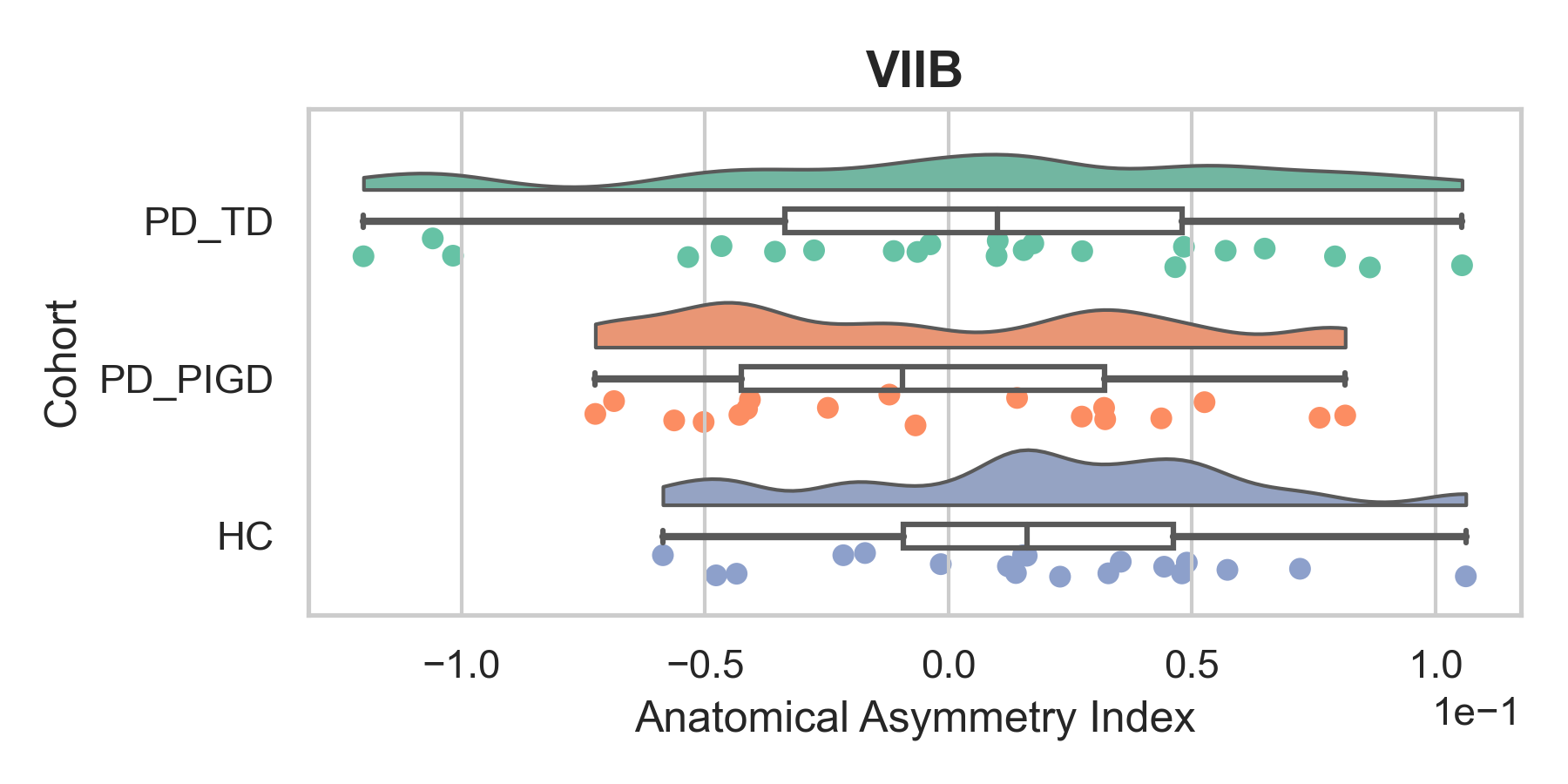

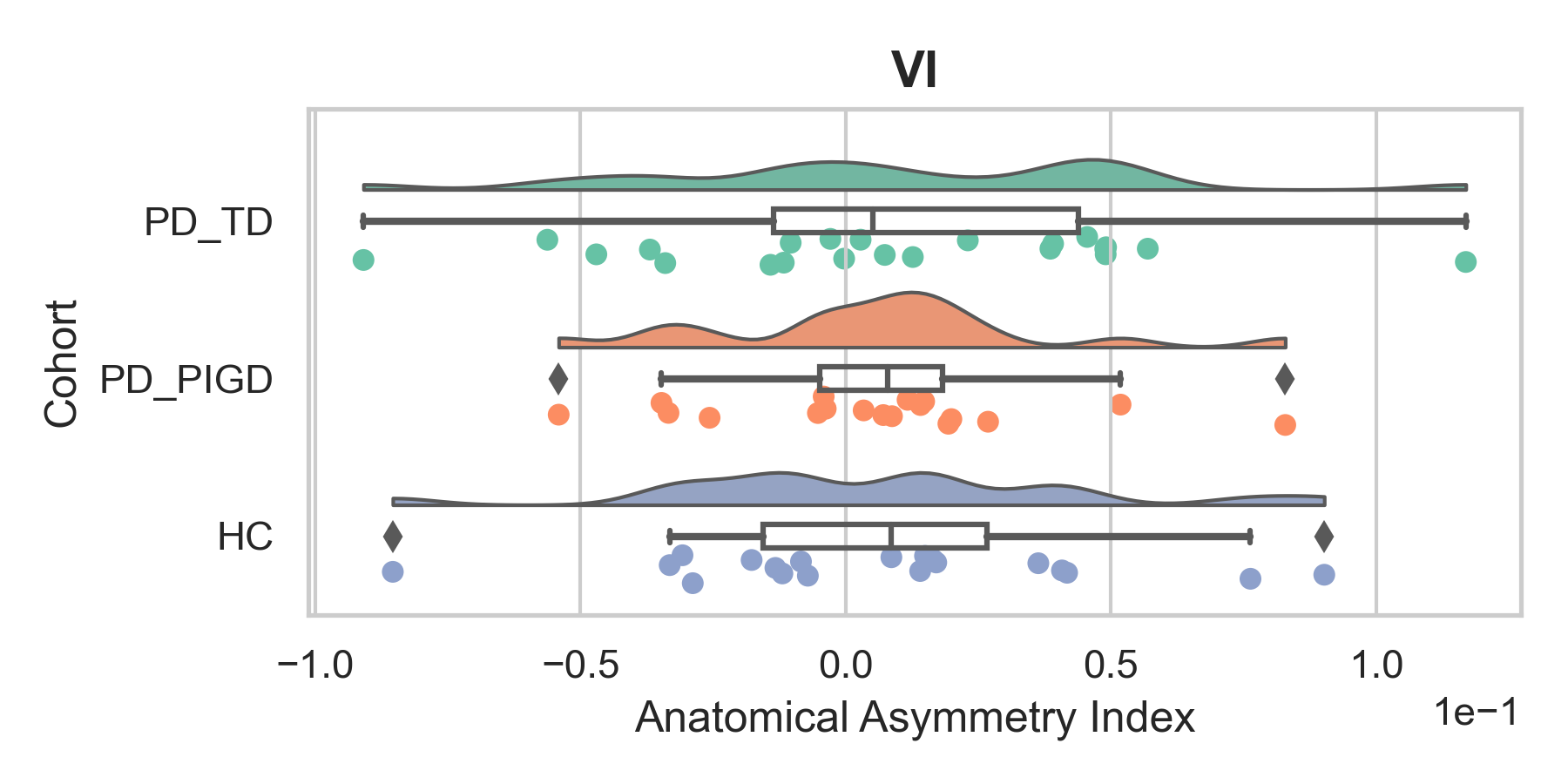

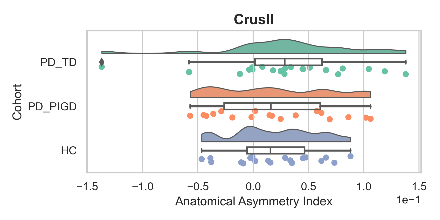

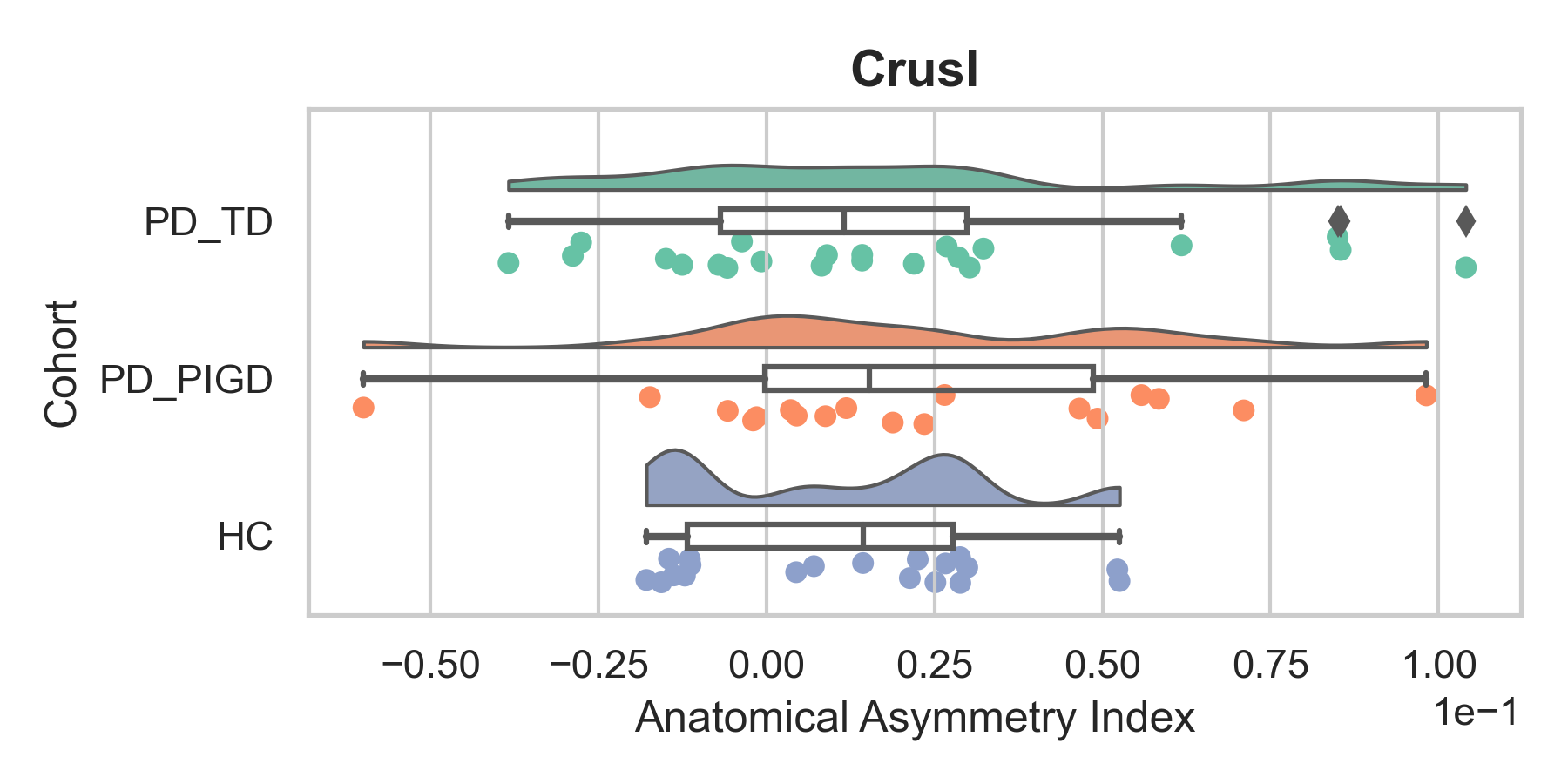

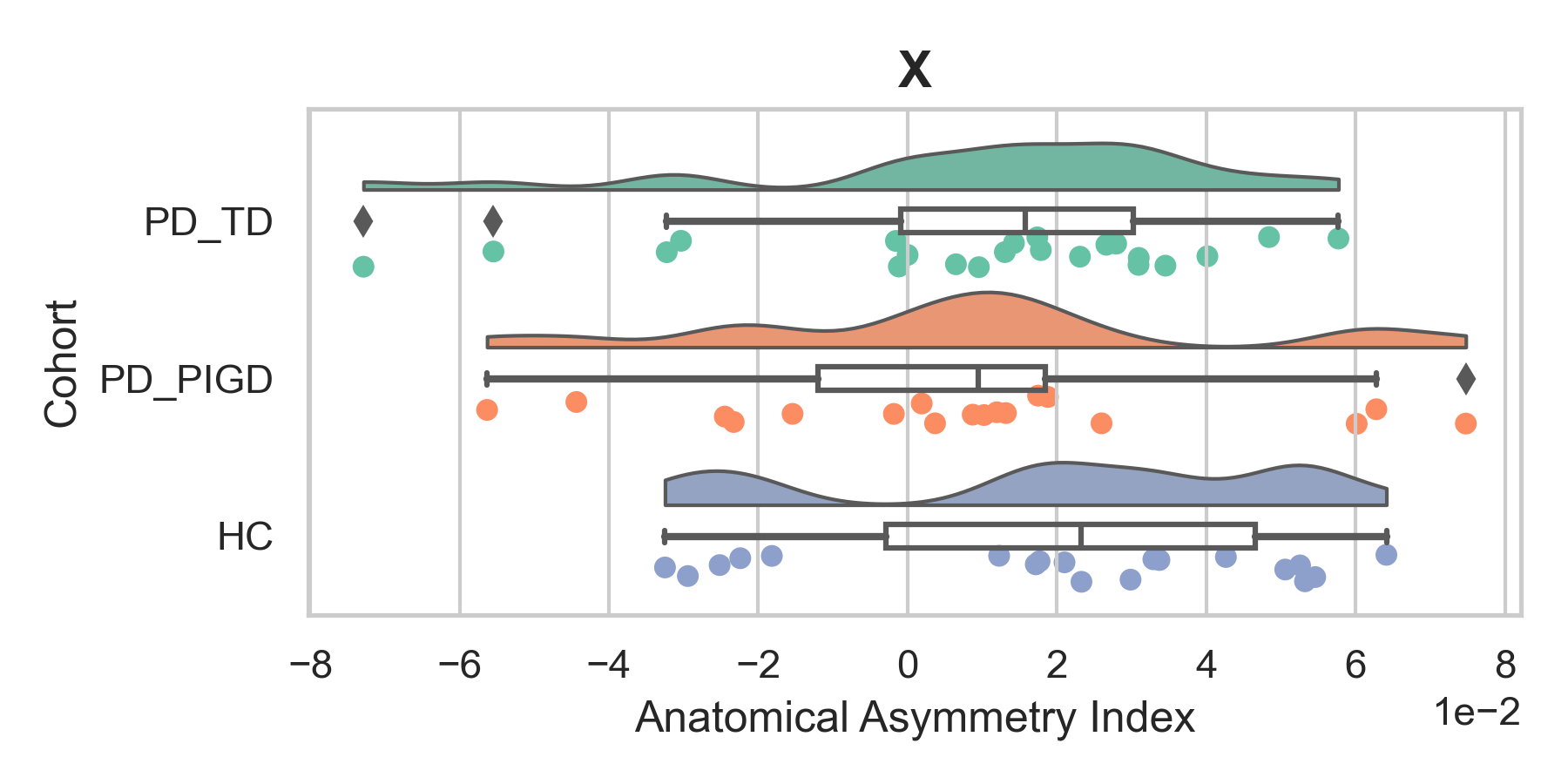

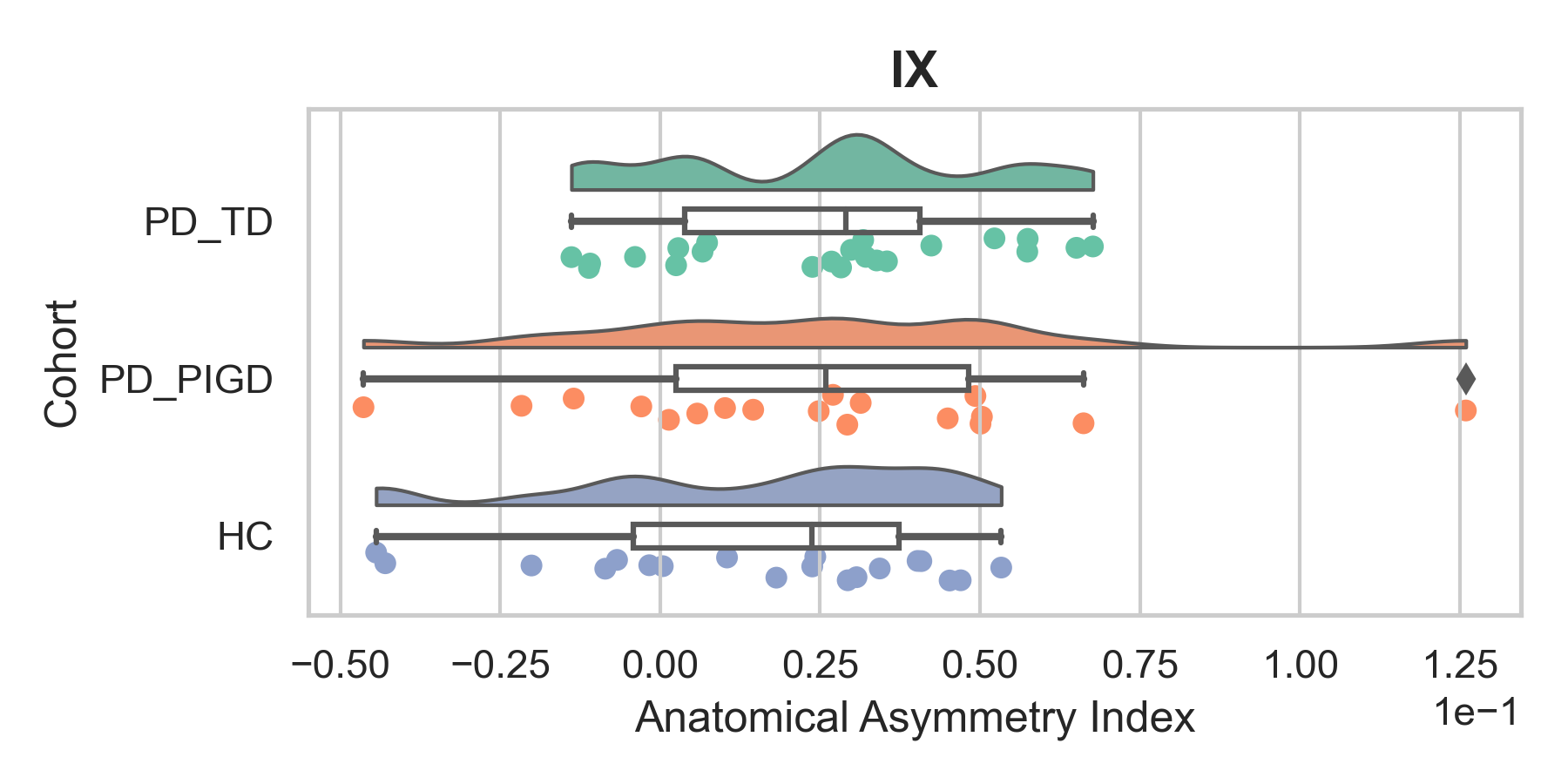


Supplementary Figure S2. Raincloud plots of anatomical asymmetry indices across cohorts. This figure visualizes the distribution of asymmetry indices for total volumes of motor-network regions across the three study groups: healthy controls (HC), tremor-dominant PD (PD-TD), and postural instability/gait disturbance PD (PD-PIGD). Each plot shows kernel density estimates (top), boxplots, and individual data points for each group. Consistent asymmetry patterns were observed across all groups, with no significant between-group differences or alignment with clinical symptom lateralization.


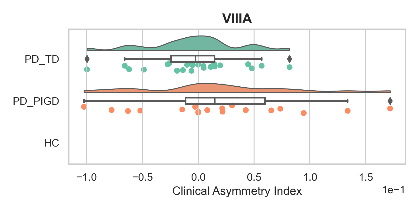

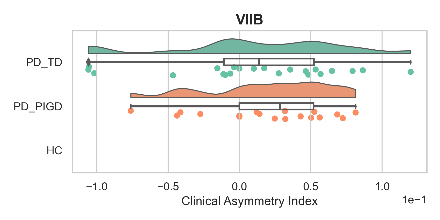

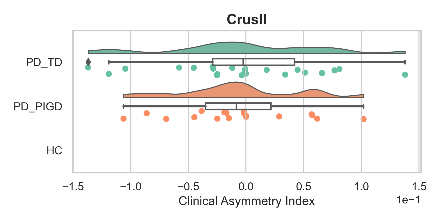

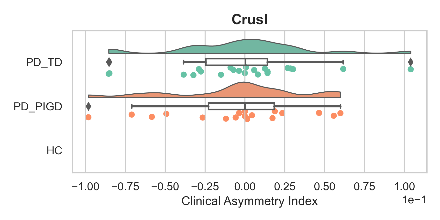

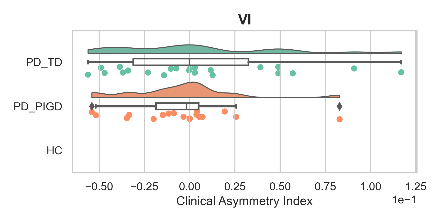

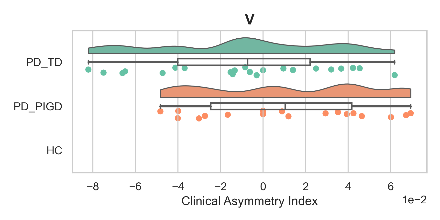

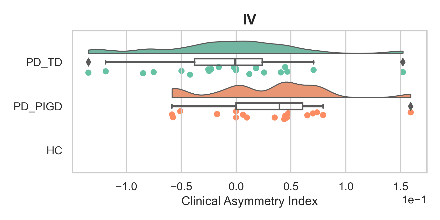

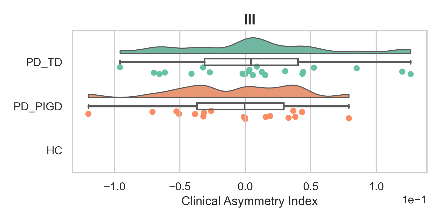

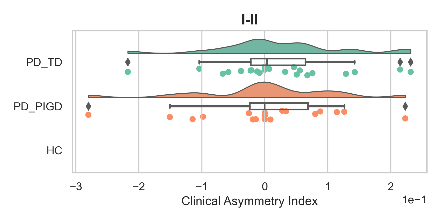

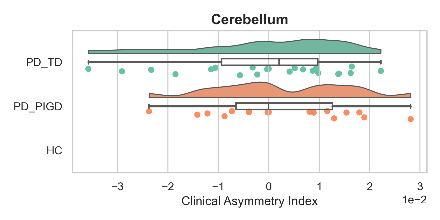

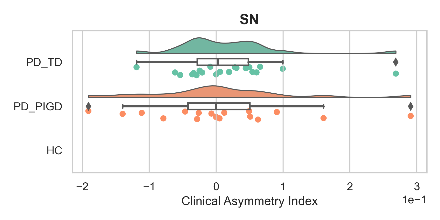

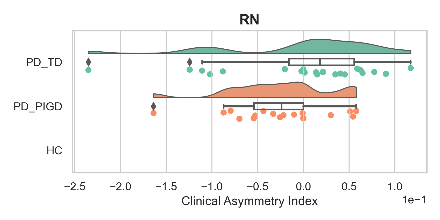

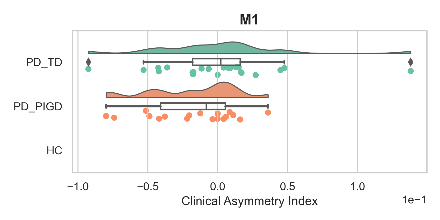

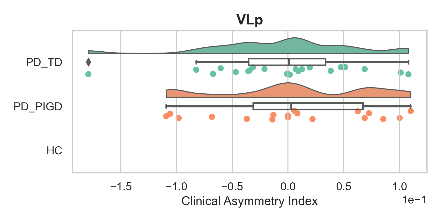

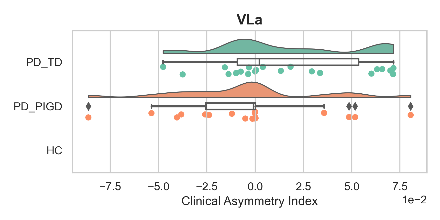

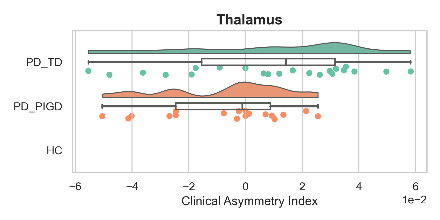

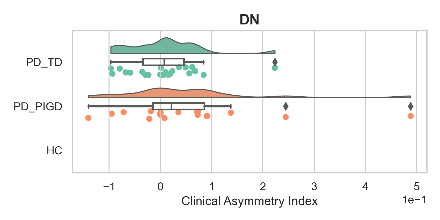

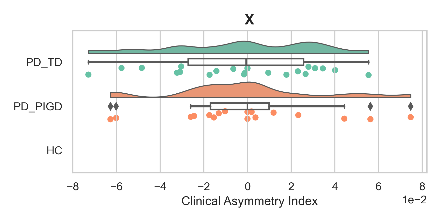

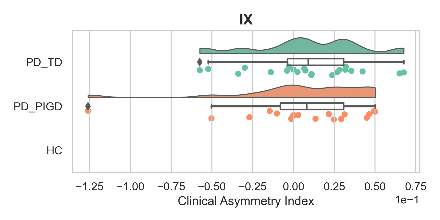

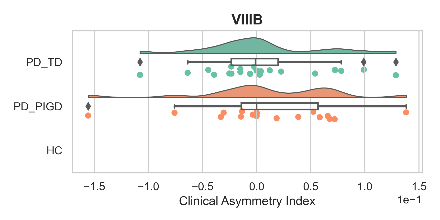


Supplementary Figure S3. Raincloud plots showing clinical asymmetry indices across motor network regions for tremor-dominant (PD-TD) and postural instability/gait disturbance (PD-PIGD) patients. Regions are grouped by anatomical location, and distributions reflect lateralized symptom severity. Each plot displays the kernel density estimate, raw data points, boxplot, and cohort mean. While the pattern of asymmetry varies across regions and subtypes, no consistent alignment with symptom lateralization was observed.


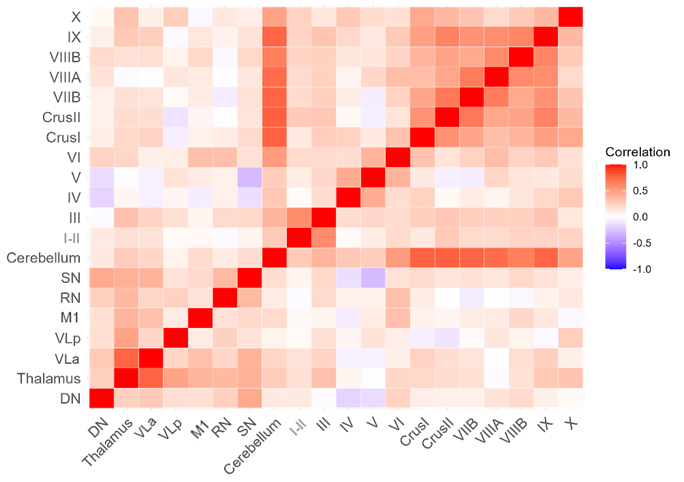


Supplementary Figure S4. Multicollinearity analysis shown by heatmap with cross-correlation values between all possible ROI pairs. Cutoff is 0.8. ROIs of the most affected side (MAS) are analyzed.

# Sensitivity Analysis: Age-Matched PD Subtype Comparison

To assess the robustness of single-ROI findings to age differences between PD motor subtypes, we repeated the PD-TD vs PD-PIGD regional analyses in an age-matched subsample. As shown in Table S1, effect directions were mostly preserved across regions. Statistical evidence was attenuated for certain regions after age matching. No new or contradictory effects emerged, indicating that the reported subtype-related volumetric patterns are not driven by age differences alone.

Table S1. Sensitivity analysis of ROI volume comparison between PD-TD and PD-PIGD (age-matched subset)

| **ROI (Side)** | **β (Main)** | **p (Main)** | **β (Age-matched)** | **p (Age-matched)** |
| --- | --- | --- | --- | --- |
| DN (MAS*) | -0.110 | 0.133 | -0.102 | 0.268 |
| DN (LAS*) | -0.171 | 0.018 | -0.125 | 0.151 |
| Thalamus (MAS) | 0.122 | 0.350 | 0.145 | 0.321 |
| Thalamus (LAS) | 0.248 | 0.024 | 0.272 | 0.032 |
| VLp (MAS) | 0.033 | 0.381 | 0.022 | 0.623 |
| VLp (LAS) | 0.051 | 0.079 | 0.043 | 0.168 |
| SN-STN (MAS) | -0.046 | 0.110 | -0.067 | 0.072 |
| SN-STN (LAS) | -0.008 | 0.769 | -0.021 | 0.499 |
| RN (MAS) | 0.002 | 0.833 | -0.002 | 0.890 |
| RN (LAS) | 0.005 | 0.679 | 0.007 | 0.616 |
| M1 (MAS) | -0.071 | 0.802 | -0.115 | 0.734 |
| M1 (LAS) | 0.160 | 0.569 | -0.190 | 0.495 |

MAS: more affected side; LAS: less affected side
